# Predicting visual impairment in type 2 diabetes using key performance indicators identified by the Black Widow and Whale Optimization Algorithms: A case study in Saudi Arabia

**DOI:** 10.1371/journal.pone.0361022

**Published:** 2026-10-21

**Authors:** Faten Al-hussein, Laleh Tafakori, Mali Abdollahian, Khalid Al-Shali

**Affiliations:** 1 Department of Mathematics and Statistics, College of Sciences, University of Jeddah, Jeddah, Saudi Arabia; 2 School of Science, RMIT University, Melbourne, Victoria, Australia; 3 Department of Medicine, King Abdulaziz University Hospital, Jeddah, Saudi Arabia; Incheon National University, KOREA, REPUBLIC OF

## Abstract

Visual impairment (VI) is a severe and prevalent complication of type 2 diabetes (T2D), particularly in high-risk populations, underscoring the critical need for early predictive strategies. A retrospective cross-sectional study was conducted to develop and validate machine learning (ML) predictive models for VI among patients with T2D in Saudi Arabia. To the best of our knowledge, this is the first study to employ WOA and BWO as feature selection techniques to identify the most informative predictors of VI among Saudi patients with T2D. Predictive models were subsequently developed and compared using both clinically recommended features and algorithmically selected features derived from WOA and BWO to determine the optimal feature set for predictive performance. Medical records of 1,000 T2D patients (555 VI [55.5%]; 445 non-VI [44.5%]) from King Abdulaziz University Hospital were analyzed. Models were developed using an 80/20 stratified train-test split, with 5-fold cross-validation performed on the training set for internal validation. Patients were divided into two groups: those with HbA1c ≥ 6.5% (full dataset) and a clinically relevant high-risk subgroup with HbA1c > 8% (reduced dataset), which incorporated 19 recommended demographic, lifestyle, and lipid profile features. Models utilizing the reduced dataset consistently achieved superior performance across all algorithms. Among them, the WOA-CatBoost model demonstrated the highest performance (Accuracy = 0.95, AUC = 0.96, 95% CI: 0.94–0.98) using only 9 features, outperforming both the BWO-Random Forest (BWO-RF) model (Accuracy = 0.92, AUC = 0.90, 95% CI: 0.87–0.93) with 10 features and the baseline Multi-Layer Perceptron (MLP) model (Accuracy = 0.91, AUC = 0.94, 95% CI: 0.91–0.97) with 13 features. Bonferroni-corrected paired *t*-*t*ests based on five-fold cross-validation accuracies (α = 0.017) confirmed a significant performance advantage of WOA-CatBoost over BWO-RF (*t* = 10.17, *p* < 0.001), suppor*t*ing WOA-CatBoost as the overall best-performing model. Key predictors identified across all three best-performing models included Hemoglobin A1c (HbA1c), body mass index (BMI), age, occupation, triglycerides (TG), type of food, and smoking. These findings demonstrate that WOA-CatBoost provides a clinically actionable, parsimonious predictive tool for VI risk stratification in T2D patients within the Saudi Arabian healthcare context.

## 1. Introduction

The global burden of type 2 diabetes (T2D) has reached alarming levels, affecting over 500 million individuals and placing significant pressure on healthcare systems, particularly in Middle Eastern countries such as Saudi Arabia [1]. T2D is associated with a broad spectrum of complications that impact multiple organ systems. Among these, visual complications are among the most debilitating, with visual impairment (VI) being one of the most severe outcomes [2,3]. Chronic hyperglycemia progressively damages the retinal microvasculature, leading to retinal hemorrhages, fluid accumulation, and macular edema, collectively resulting in a gradual or abrupt decline in visual function [2]. Epidemiological evidence indicates that approximately 80% of individuals with a diabetes duration of 15–20 years experience some form of VI [4]. Globally, VI remains a major public health concern, affecting approximately 2.2 billion people, with an estimated 3.9 million cases attributed to diabetic retinopathy [5]. Given this vast scale, VI leads to a reduction in a person’s ability to work and perform daily activities, leading to higher healthcare costs and a poorer quality of life among people with diabetes [6].

Previous studies have documented a substantial and accelerating increase in the global burden of VI attributable to diabetic retinopathy over the past three decades [7–9]. Epidemiological studies across diverse populations have identified a common set of risk factors associated with VI among patients with T2D. A Chinese cohort study of 1,633 patients with diabetes conducted between 2015 and 2018 found that 11.8% presented with VI at baseline, with older age, female sex, lower educational attainment, rural residence, and multiple comorbidities identified as key risk factors [10]. Similarly, a study conducted in Ethiopia among 322 patients with T2D reported a VI prevalence of 37.6%, with older age, diabetes duration exceeding five years, exclusive insulin therapy, and poor glycaemic control (elevated HbA1c) identified as significant predictors [11]. Furthermore, a prospective cohort study in a multi-ethnic Asian population identified older age, lower income, and limited education as the leading risk factors for incident VI, while diabetes and hypertension were additional contributing factors [12]. In South Korea, a study applying machine learning models to predict diabetic retinopathy (DR) among 14,694 patients demonstrated that XGBoost achieved an AUROC of 0.82, with HbA1c, glucose variability, dyslipidaemia, and cardiovascular comorbidities emerging as key predictors [13].

HbA1c is widely established as a reliable biomarker for glycaemic control in T2D and has been consistently linked to the severity of diabetes-related visual complications across multiple populations [14–17]. Higher HbA1c levels are significantly associated with increased incidence and progression of DR, with evidence suggesting that even modest reductions in HbA1c confer meaningful protection against vision-threatening complications [15,18]. However, while HbA1c reliably identifies patients with poor glycaemic control, it functions as a population-level risk indicator rather than a patient-specific predictive tool, underscoring the need for multivariable predictive models that integrate HbA1c alongside other clinical features to accurately identify which individual patients will develop VI.

Previous studies showed that the Whale Optimization Algorithm (WOA) and Black Widow Optimization (BWO) have demonstrated strong performance in feature selection tasks, particularly in high-dimensional medical data environments. Compared to conventional metaheuristic approaches such as Particle Swarm Optimization (PSO) and Genetic Algorithms (GA), WOA offers superior convergence behaviour and a well-balanced exploration–exploitation trade-off [19,20], while BWO achieves faster convergence and effective avoidance of local optima through its biologically inspired mechanisms [21,22]. These algorithms have demonstrated growing adoption across diverse medical applications, including brain tumour diagnosis [23], gene selection [24], COVID-19 prediction [25], and disease diagnosis tasks spanning Parkinson’s disease and chronic liver disease [26].

These theoretical justifications are further supported by multiple applied studies, that have demonstrated the practical effectiveness of both WOA and BWO across diverse medical prediction tasks [27–30]. For example, in heart disease prediction, WOA-based feature selection applied across five datasets and multiple classifiers, consistently improved predictive performance, achieving accuracies exceeding 99% and improved predictive performance, achieving accuracies exceeding 99% alongside improvements in precision, recall, F1-score, and AUC [31]. Additionally, a WOA-based ensemble classifier surpassed individual models, achieving 83% accuracy and a true positive rate of 0.94 in diabetes prediction [32]. More directly relevant to the present study, WOA has also demonstrated strong performance in ophthalmic prediction tasks. In eye disease classification, integrating WOA with deep learning achieved 99.1–99.4% accuracy and high Kappa scores across DR, glaucoma, and cataract classification tasks [33], supporting its relevance to diabetes-related ocular risk modelling. Likewise, BWO has shown promising performance in medical feature selection applications, with a BWO–AdaBoost integration achieving 86.1% prediction accuracy [34], and a binary BWO–SVM model attaining 91.0% accuracy with a reduced feature subset, outperforming alternative metaheuristic-based approaches [35]. Although these studies address different clinical outcomes, they consistently demonstrate the ability of WOA and BWO to improve predictive performance while reducing model complexity.

In Saudi Arabia, several studies have examined diabetes-related ocular complications among T2D patients, predominantly focusing on DR risk factors across different regions. A study in Abha (n = 401) reported a DR prevalence of 36.4%, identifying younger age at onset, longer diabetes duration, insulin therapy, poor glycaemic control (HbA1c > 9%), hypertension, neuropathy, and nephropathy as significant predictors [36]. Similarly, a study in Makkah (n = 251) reported a higher DR prevalence of 54.6%, with diabetes duration of 11–16 years, poor medication adherence, uncontrolled HbA1c (≥7%), and higher BMI among the key risk factors identified through multivariate analysis [37]. In Medina (n = 130), HbA1c ≥ 7% and diabetes duration ≥11 years were found to significantly increase the odds of DR (OR = 6.9 and 5.2, respectively) [16], while a Riyadh-based study (n = 428) demonstrated markedly higher DR prevalence in the high-risk HbA1c group (88.1%) compared to the low-risk group (22.1%) [18]. In Qassim (n = 106), higher HbA1c, longer disease duration, insulin use, and macular oedema were associated with greater DR severity [14]. More recently, a machine learning study in Almajmaah (n = 327) demonstrated that a Random Forest classifier achieved 86% accuracy in predicting DR, with HbA1c, diabetes duration, BMI, age at onset, and insulin use identified as the most influential predictors [38].

Although existing studies have advanced the understanding of diabetes-related ocular complications, the early prediction of VI among T2D patients remains challenging. Several limitations remain in the current literature, including: (i) a predominant focus on DR rather than broader VI outcomes [36–38]; (ii) reliance on conventional statistical or single-biomarker approaches, including HbA1c-based risk stratification, which primarily reflect population-level risk rather than patient-specific prediction and may be less effective in capturing complex, non-linear interactions among demographic, metabolic, and clinical factors [13,16,18]; (iii) limited application of advanced machine learning methods, particularly nature-inspired feature selection techniques [38]; and (iv) the absence of locally validated predictive models for Saudi Arabian populations, limiting the generalizability of international findings to the regional healthcare context [16,18,36–38].

To address these gaps, the present study develops an internally validated machine-learning framework for identifying VI among patients with T2D using locally collected Saudi clinical data. The novelty of this study is fourfold. First, it focuses on visual impairment among Saudi patients with T2D, whereas many previous studies have primarily examined diabetic retinopathy or individual clinical risk factors. Second, it utilizes clinically recommended predictors and predictors selected using two nature-inspired feature selection methods, namely the Whale Optimization Algorithm (WOA) and Black Widow Optimization (BWO). Third, it systematically assesses the predictive performance of 11 machine-learning classifiers, including Adaptive Boosting (AdaBoost), Logistic Regression (LR), Support Vector Machine (SVM), Decision Tree (DT), K-Nearest Neighbors (KNN), Random Forest (RF), Multi-Layer Perceptron (MLP), Naïve Bayes (NB), Extreme Gradient Boosting (XGBoost), Categorical Boosting (CatBoost), and Gradient Boosting (GraBoost), both with and without nature-inspired feature selection, under a consistent validation framework. Fourth, it evaluates model performance in both HbA1c levels greater than or equal to 6.5% (full dataset), and a high-risk subgroup with HbA1c levels greater than 8% (reduced dataset), thereby improving clinical interpretability. We hypothesize that integrating WOA and BWO based feature selection techniques with machine learning classifiers will enhance predictive performance compared with baseline models while identifying a parsimonious set of clinically relevant predictors for VI. Consequently, this study provides a robust methodological framework for VI prediction and establishes a foundation for future external validation and clinical implementation within Saudi healthcare settings.

## 2. Materials and methods

This section provides a comprehensive overview of the dataset analysis process and the adopted methodology, including procedures for feature selection, model training, and performance evaluation, which were utilized to classify cases into two groups: VI and non-VI. Fig 1 illustrates the methods used in the study.

**Fig 1:**
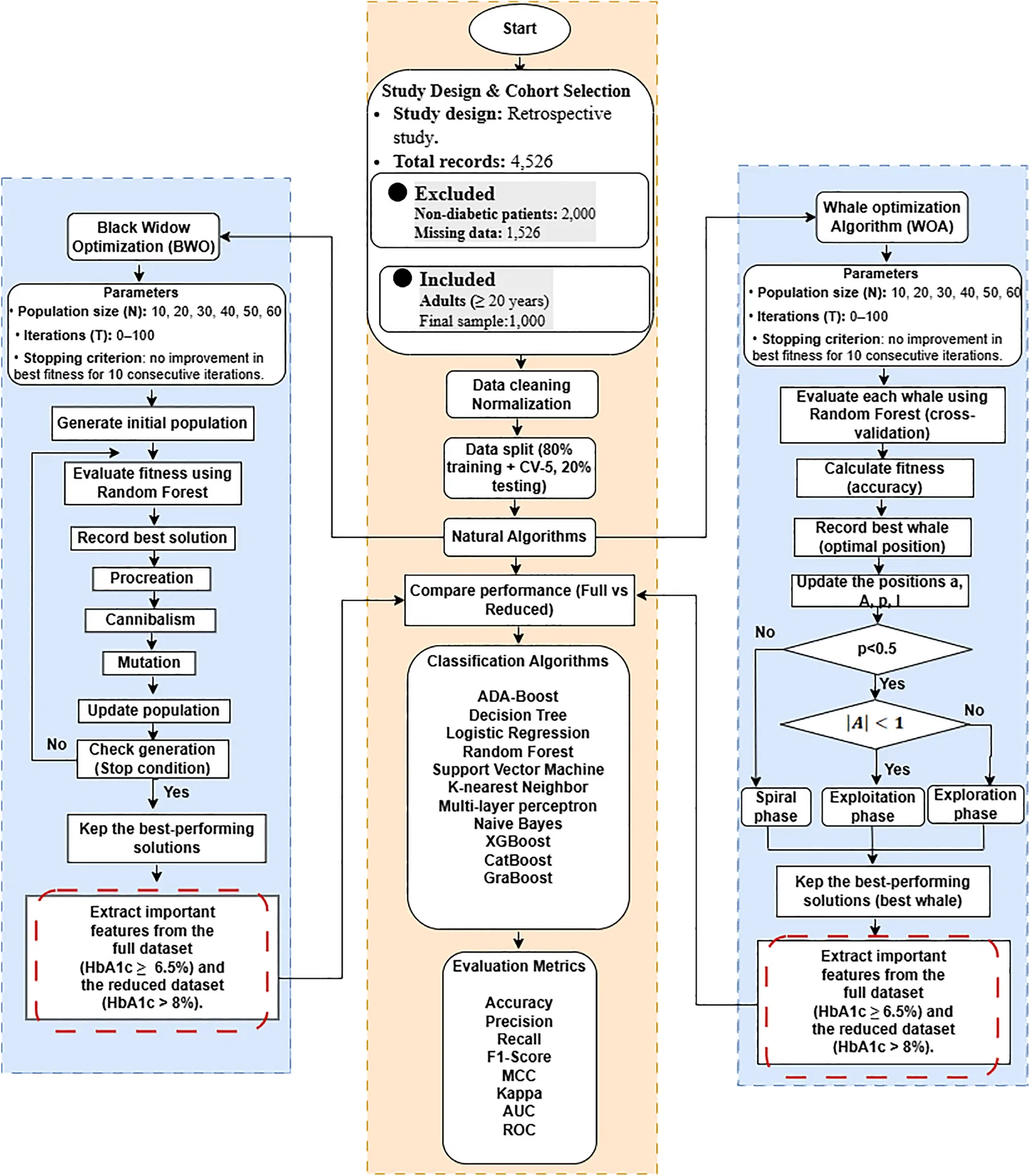
Research methodology.

### 2.1 Data collection

The research draws upon anonymized medical records of 4,526 patients aged 20 years or older with a confirmed diagnosis of T2D who received care at King Abdulaziz University Hospital (KAUH) in Jeddah, Saudi Arabia, between 01/01/2018 and 31/12/2022. The dataset had been previously compiled and validated by qualified healthcare professionals to ensure data reliability and accuracy. For analysis, patients were divided into two categories: those identified with VI based on the ophthalmologic diagnosis recorded in the patient’s medical history, and those without. The study received ethical approval from both the Human Research Ethics Committee at RMIT University in Australia and the Research Ethics Committee at King Abdulaziz University Hospital. Given the retrospective cross-sectional design of the study and the exclusive use of previously collected, de-identified medical data, the requirement for informed consent was formally waived by both ethics boards. Access to the data was granted on 19/11/2023, following official ethical approval.

To ensure data quality, 2,000 non-diabetic records were first excluded from the original dataset of 4,526 records, leaving 2,526 patients with T2D. The remaining records of patients with T2D then underwent a series of preprocessing steps. Variables with more than 90% missing data were excluded, and variables exhibiting near-zero variance, defined as those in which at least 90% of patients shared the same value, were subsequently removed. These preprocessing steps reduced the number of variables from 35 to 19. Records with substantial missing data that precluded reliable imputation were then excluded, resulting in a final analytical cohort of 1,000 patients with T2D. For the remaining records, missing values were handled using mean imputation for continuous variables and mode imputation for categorical variables. To ensure statistical robustness, sample adequacy was assessed using the Events-Per-Variable (EPV) criterion, yielding an EPV ratio of 29.2, substantially exceeding the recommended minimum threshold of 10. Normality of continuous variables was evaluated using the Shapiro–Wilk test, with all variables satisfying the normality assumption (p > 0.05). Multicollinearity was assessed using the Variance Inflation Factor (VIF), with all predictors remaining within acceptable limits (maximum VIF = 4.12; threshold < 5.0). To control for potential inflation of Type I error arising from multiple univariate comparisons in Table 1, Bonferroni correction was applied, yielding an adjusted significance threshold of α = 0.0026 (0.05/19).

**Table 1:** Description and comparison of feature frequencies between Vision Impairment (VI) and Non-Vision Impairment (Non-VI) groups.

Variables	Total (n = 1000) (%)	VI n = 555	Non-VI n = 445	p-value Comparing VI and Non-VI
**Gender**				0.0082
Female	446(44.56%)	236(52.91%)	210(47.09%)	
Male	554(55.44%)	319(57.58%)	235(42.42%)	
**Age**				<0.001
20-39	42(4.20%)	25(59.52%)	17(40.48%)	
40-59	293(29.30%)	159(54.27%)	134(45.73%)	
60-79	530(53.00%)	295(55.66%)	235(44.34%)	
80 and above	135(13.50%)	76(56.29%)	59(43.70%)	
**BMI**				0.0261
Underweight	22(2.20%)	10(45.45%)	12(54.55%)	
Normal weight	184(18.47%)	40(21.74%)	144(78.26%)	
Overweight	337(33.77%)	197(58.46%)	140(41.54%)	
Obese	457(45.70%)	308(67.39%)	149(32.60%)	
**Marital Status**				0.9124
Married	818 (82.00%)	431(52.69%)	387(47.31%)	
Not Married	182(18.20%)	124(68.13%)	58(31.87%)	
**Nationality**				0.5767
Saudi Arabia	639 (63.90%)	353(55.24%)	286(44.76%)	
Not Saudi Arabia	361 (36.10%)	202(55.96%)	159(44.04%)	
**Occupation**				<0.001
Employed	288 (28.80%)	164(56.94%)	124(43.06%)	
Not Employed	712 (71.20%)	391(54.92%)	321(45.08%)	
**Smoking**				<0.001
Yes	869 (86.90%)	470(54.09%)	399(45.91%)	
No	131 (13.10%)	85(64.89%)	46(35.11%)	
**Physical Activity**				0.9327
Yes	871 (87.10%)	458(52.58%)	413(47.42%)	
No	129 (12.90%)	97(75.19%)	32(24.81%)	
**Type of Food**				<0.001
Healthy	351 (35.10%)	228(64.96%)	123(35.04%)	
Non healthy	649 (64.90%)	327(50.38%)	322(49.61%)	
**Hypertension**				<0.001
Yes	782 (78.00%)	411(52.56%)	371(47.44%)	
No	218 (21.80%)	144(66.06%)	74(33.94%)	
**WBC**				0.5393
Low	46 (4.60%)	25(54.35%)	21(45.65%)	
Good	458 (45.80%)	268(58.52%)	190(41.48%)	
High	496 (49.60%)	262(52.82%)	234(47.18%)	
**HDL**				0.0418
Low	585 (58.50%)	339(57.95%)	246(42.05%)	
Good	224 (22.40%)	129(57.59%)	95(42.41%)	
High	191 (19.10%)	87(45.55%)	104(54.45%)	
**TC**				0.0711
Normal	223 (22.30%)	83(37.22%)	140(62.78%)	
Moderately high	206 (20.60%)	98(47.57%)	108(52.43%)	
High	571 (57.10%)	374(65.50%)	197(34.50%)	
**TG**				<0.001
Normal	198 (19.80%)	83(41.92%)	115(58.08%)	
Moderately high	206 (20.60%)	116(56.31%)	90(43.69%)	
High	596 (59.60%)	356(59.73%)	240(40.27%)	
**Vitamin- D**				<0.001
Deficient	478 (47.70%)	387(80.96%)	91(19.04%)	
Insufficient	231 (23.10%)	73(31.60%)	158(68.40%)	
Sufficient	291 (29.10%)	95(32.65%)	196(67.35%)	
**Ferritin**				0.0140
Deficient	581(58.10%)	311(53.53%)	270(46.47%)	
Insufficient	230(23.00%)	145(63.04%)	85(36.96%)	
Sufficient	189(18.90%)	99(52.38%)	90(47.62%)	
**Systolic blood pressure**				0.9516
Low	166(16.58%)	16(9.64%)	150(90.36%)	
Normal	381(38.10%)	163(42.78%)	218(57.22%)	
High	453(45.25%)	376(83.00%)	77(17.00%)	
**Diastolic blood pressure**				0.0520
Low	668(66.80%)	375(56.14%)	293(43.86%)	
Normal	181(18.08%)	106(58.56%)	75(41.44%)	
High	151(15.09%)	74(49.01%)	77(50.99%)	
**HbA1c**				<0.001
Control (<7%)	179(17.90%)	4(2.23%)	175(97.76%)	
Uncontrol (≥7%−8%)	281(28.1%)	183(65.12%)	98(34.87%)	
Poor (>8%)	540(54.00%)	368(68.15%)	172(31.85%)	

HDL: High-Density Lipoprotein level, TC: Total Cholesterol Level, WBC: White Blood Cells, TG: Triglycerides Level, BMI: Body Mass Index. Percentages were calculated using the following formulas: Total = n/1000; VI = (number of VI patients within each category)/(total number of patients in that category); Non-VI = (number of Non-VI patients within each category)/(total number of patients in that category).

### 2.2 Classification of key performance indicators (KPIs) and comparison between vision impaired and non-impaired groups

In this study, the target variable indicating the presence of VI was coded as 0 (absent) or 1 (present). The key performance indicators (KPIs) were categorized into three main groups: demographic characteristics (gender, nationality, age, marital status, and body mass index (BMI)), lifestyle factors (smoking status, occupation, hypertension, type of food, and physical activity), and clinical health indicators (total cholesterol (TC), diastolic blood pressure (DBP), ferritin, white blood cell (WBC), hemoglobin A1c (HbA1c), high-density lipoprotein (HDL), systolic blood pressure (SBP), vitamin-D, and triglycerides (TG).

BMI classifications were determined according to the standards set by the World Health Organization (WHO) and established BMI guidelines [39,40]. Four categories were applied: underweight (BMI < 18.5 kg/m²), normal weight (18.5–24.9 kg/m²), overweight (25.0–29.9 kg/m²), and obese (≥30.0 kg/m²). HDL cholesterol values were interpreted based on gender-specific thresholds. Levels were considered low if below 40 mg/dL for males or 50 mg/dL for females, adequate between 40–59 mg/dL for males and 50–59 mg/dL for females, and high when reaching 60 mg/dL or above [41]. TG were categorized into three ranges: normal (<149 mg/dL), moderately elevated (150–199 mg/dL), and high (≥200 mg/dL) [41]. TC levels were assessed as desirable when below 200 mg/dL, borderline high between 200–239 mg/dL, and high if 240 mg/dL or more [41]. Vitamin-D status was evaluated using clinical reference thresholds, where values below 30 ng/mL indicated deficiency, levels between 30–50 ng/mL reflected insufficiency, and levels of 50 ng/mL or higher were considered sufficient [42]. Ferritin levels were interpreted using gender-specific reference ranges, with normal values of 24–336 µg/L in females and 11–307 µg/L in males. Values below these ranges could indicate iron deficiency, while elevated levels might reflect iron overload or an underlying inflammatory condition [43]. According to WHO criteria, an HbA1c level of 6.5% or higher is used to diagnose diabetes, while levels below 6.5% are considered non-diabetic. In clinical settings, cases with an HbA1c level of 7% or less are considered controlled, whereas levels above 7% are classified as uncontrolled [44].

Table 1 summarizes the demographic and clinical characteristics of 1,000 patients with T2D, including 555 patients with VI (55.5%) and 445 without VI (44.5%). After Bonferroni correction for 19 univariate comparisons (α = 0.0026), age, occupation, smoking, type of food, hypertension, HbA1c, triglycerides, and vitamin D remained significantly associated with VI. Gender, BMI, HDL, and ferritin showed nominally significant associations in the univariate analysis (p < 0.05) but did not remain statistically significant after Bonferroni correction. Marital status, nationality, physical activity, total cholesterol, WBC, systolic blood pressure, and diastolic blood pressure were not significantly associated with VI after adjustment. Among the significant clinical variables, HbA1c showed a particularly strong association with VI: among patients with HbA1c < 7% (n = 179), only four patients had VI (2.23%), whereas VI prevalence was substantially higher among patients with HbA1c > 8% (68.15%).

Fig 2 displays the distribution of VI across three categories of HbA1c: controlled (<7%), uncontrolled (≥7–8%), and poorly controlled (>8%). The visual pattern highlights a strong inverse relationship between glycemic control and vision health. In the controlled group (HbA1c < 7%), VI was extremely rare, affecting only 2.2% of individuals within this group, while 97.8% did not exhibit any VI. In contrast, the uncontrolled group (HbA1c ≥ 7–8%) showed a sharp increase in visual complications, with 65.1% of individuals in this category experiencing VI. Moreover, in the poorly controlled group (HbA1c > 8%), 68.2% of the group had VI. Among the three HbA1c categories, individuals with poor control (HbA1c > 8%) accounted for the highest number of VI cases (n = 368). These findings indicate a strong association between poor glycemic control and VI, emphasizing the need to focus on this high-risk group. Previous studies have demonstrated the impact of HbA1c levels on visual health [15–18], and this study specifically highlighted this subgroup (reduced dataset) due to the high prevalence of VI observed among its members. Fig 3 shows weak or no correlations among the variables before feature selection.

**Fig 2:**
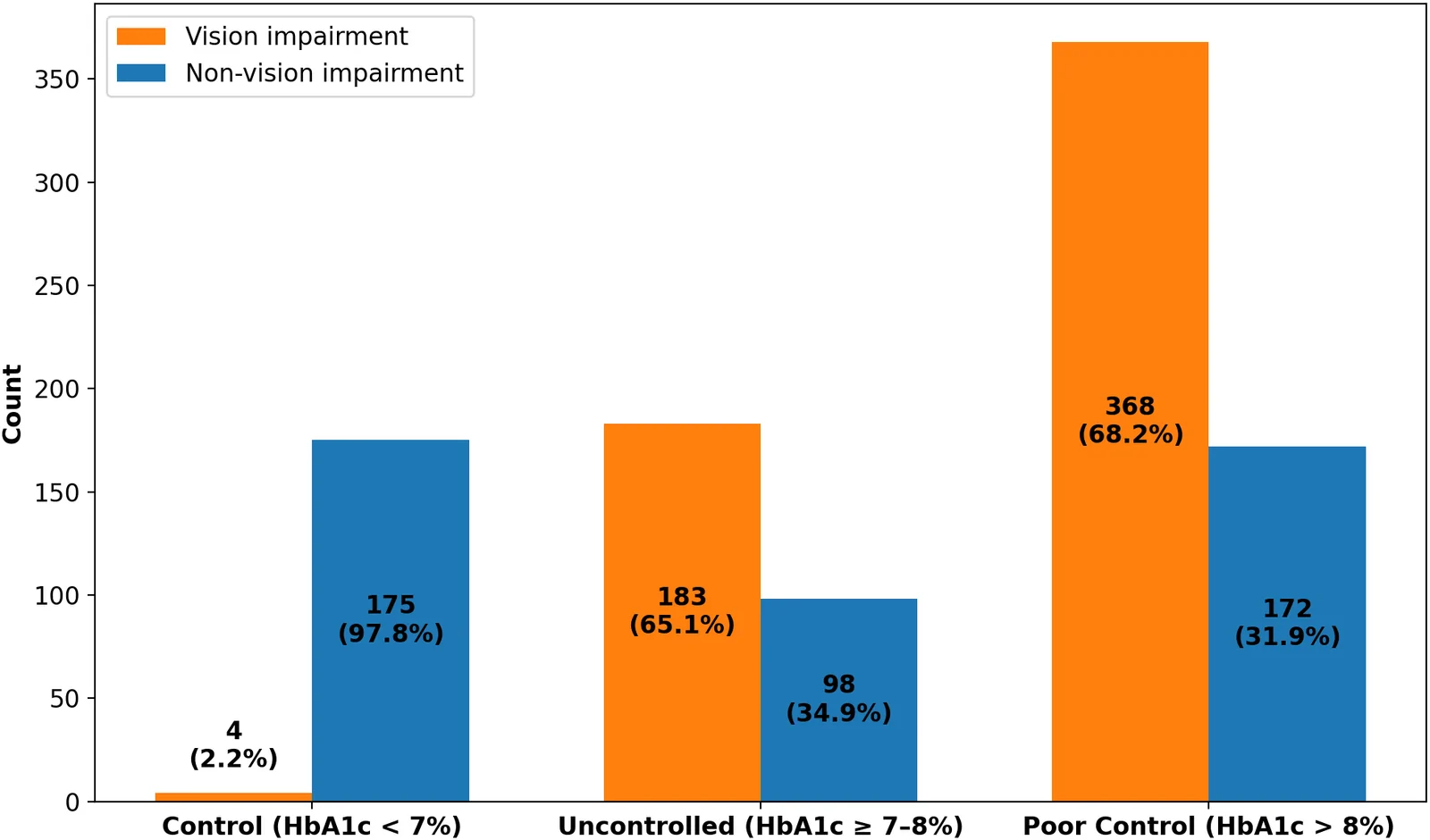
Distribution of HbA1c categories within each vision group.

**Fig 3:**
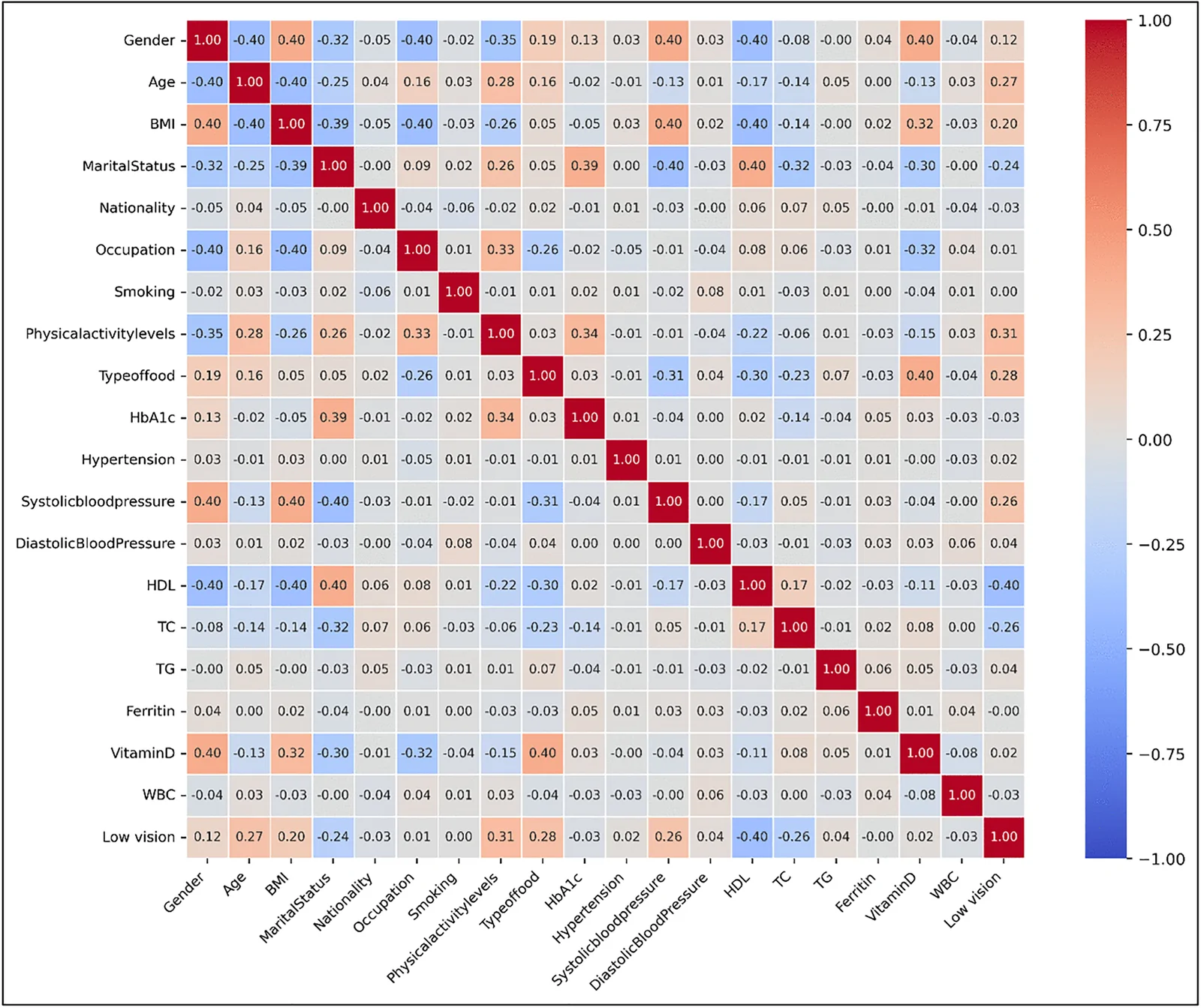
Correlation Heatmap visualization before feature selection.

## 3. Model development

The proposed framework aims to enhance classification performance and reduce the number of variables through the use of two nature-inspired optimization algorithms, namely BWO and WOA. The process begins with data preprocessing, which includes normalization and an 80/20 train-test split to ensure robust model evaluation. To preserve class proportions across all data partitions and account for the mild class imbalance observed in the dataset (555 VI vs. 445 non-VI; 55.5% vs. 44.5%), stratified sampling was applied during both the 80/20 train-test split and the 5-fold cross-validation. Additionally, class imbalance was addressed by applying the class_weight = ‘balanced’ parameter in applicable classifiers to reduce potential model bias. A 5-fold cross-validation was employed on the training set to ensure robust evaluation and prevent overfitting. Fig 1 presents the overall workflow of the methodological framework.

Feature selection is performed independently using both BWO and WOA. The BWO algorithm utilises evolutionary mechanisms, including procreation, cannibalism, and mutation, to iteratively refine feature subsets through a Random Forest (RF)-based fitness evaluation. In parallel, the WOA algorithm optimizes feature subsets through spiral updating, exploitation, and exploration phases, guided by the accuracy of cross-validated RF classifiers. Following the optimal feature selection process, key performance indicators (KPIs) are identified for two patient groups: the all-patient cohort with HbA1c ≥ 6.5%, referred to as the full dataset, and the high-risk subgroup with HbA1c > 8%, referred to as the reduced dataset. These KPIs are subsequently integrated into 11 classification algorithms to determine the best-performing model under each optimization strategy, including Adaptive Boosting (AdaBoost), Logistic Regression (LR), Support Vector Machine (SVM), Decision Tree (DT), K-Nearest Neighbors (KNN), Random Forest (RF), Multi-Layer Perceptron (MLP), Naïve Bayes (NB), Extreme Gradient Boosting (XGBoost), Categorical Boosting (CatBoost), and Gradient Boosting (GraBoost). The same classification models are additionally applied to the baseline dataset for comparison purposes. Model performance is evaluated using accuracy, precision, recall, F1-score, Matthews Correlation Coefficient (MCC), Cohen’s Kappa (κ), and Area Under the Curve (AUC). All analyses, including data preprocessing and model development, were implemented in Python 3.12 using Jupyter Notebook, and the associated code is publicly available at https://github.com/fatenalhussains/Predicting-VI-in-T2D-Using-KPIs-Identified-by-the-BWO-and-WOA-A-Case-Study-in-Saudi-Arabia.git.

### 3.1 Nature-inspired optimization algorithms

#### 3.1.1 Black Widow Optimization (BWO).

The Black Widow Optimization (BWO) algorithm is a population-based metaheuristic inspired by the mating and survival strategies of black widow spiders [22]. Each candidate solution is represented as a widow vector of length equal to the number of decision variables (N_var_), encoded as a binary string where 1 indicates feature inclusion and 0 indicates exclusion. Solution quality is measured by a fitness function f(x). The algorithm proceeds through three sequential operators: (i) procreation, in which pairs of parents generate offspring via a linear combination controlled by a random coefficient α [22,35]. (ii) cannibalism, in which weaker individuals are eliminated at three levels (parent–parent, sibling–sibling, and offspring–mother), preserving only the fittest candidates [22,45]. (iii) mutation, in which a subset of solutions undergoes random element swaps to maintain population diversity [22,46]. The three governing parameters procreation rate (Pp), cannibalism rate (Cr), and mutation rate (Pm) regulate reproduction proportion, elimination rate, and mutation frequency, respectively [22,46,47]. Convergence is determined by one of three stopping criteria: reaching a maximum iteration count, observing no improvement in the best fitness value over successive iterations, or attaining a predefined accuracy threshold. Mathematical derivations and pseudocode are provided in S1 File.

#### 3.1.2 Whale Optimization Algorithm (WOA).

The Whale Optimization Algorithm (WOA) is a bio-inspired metaheuristic modelled after the bubble-net hunting strategy of humpback whales [20]. The algorithm operates through two core mechanisms: encircling prey, in which candidate solutions converge toward the best-known position using linearly shrinking coefficient vectors $\vec{A}$ and $\vec{C}$; and spiral updating, in which positions are updated along a helix-shaped path toward the current best solution [20,48]. At each iteration, a random probability p determines whether a whale follows the encircling or spiral trajectory. For exploration $\left(\vec{A}>\text{1}\right)$, positions are updated relative to a randomly selected whale ${\vec{X}}_{rand}\;$ rather than the global best, enabling escape from local optima [48,49]. This dual exploitation exploration balance has demonstrated strong performance in feature selection and classification tasks [20,50]. Mathematical derivations are provided in S2 File.

### 3.2 Machine learning techniques

The previous work by the authors [51] provided a comprehensive and detailed explanation of some machine learning models in terms of their theoretical foundations and operational mechanisms in the methodology section, including Logistic Regression (LR), Support Vector Machine (SVM), Decision Tree (DT), K-Nearest Neighbors (KNN), Random Forest (RF), Naïve Bayes (NB), Extreme Gradient Boosting (XGBoost), Adaptive Boosting (AdaBoost), and Gradient Boosting (GraBoost) as summarized in S1 Table. Since these models were thoroughly discussed and evaluated in the previous work, we have included a brief description in the attached supplementary materials. We will provide a detailed description of the methodology for the remaining models in this study, namely Categorical Boosting (CatBoost) and Multilayer Perceptron (MLP).

#### 3.2.1 Categorical Boosting (CatBoost).

CatBoost is a gradient boosting algorithm specifically designed to handle categorical features efficiently, eliminating the need for traditional preprocessing methods such as one-hot encoding [52,53]. Given a dataset $D=\left\{(Z_{i},\;y_{i})\right\}\;i=\text{1},\ldots ,m$, where each $Z_{i}=\left(z_{i\text{1}},\;z_{i\text{2}},\;\ldots ,z_{in}\right)\;\in R^{n}$ represents a feature vector of dimension n and $y_{i}\in R$ denotes the corresponding target variable, the goal is to approximate a predictive function $F:\;R^{n}\to R$ that minimizes the expected loss defined as J(F)=E[ℓ(y,F(Z)], with 𝓁(·,·) being a smooth loss function such as the mean squared error for regression or the log-loss for classification tasks. In the gradient boosting framework, the model is constructed iteratively as a sequence of approximations, where at the $k^{th}$ iteration the function is updated according to the following expression:


$$\begin{array}{c} F^{k}(Z)=F^{k-\text{1}}(Z)+\;\eta_{k}h^{k}(Z). \end{array}$$

Here, $h^{k}(Z)$ is a weak learner, typically a binary decision tree selected from a hypothesis space H and $\eta_{k}$ denotes the learning rate controlling the contribution of each new tree. The weak learner is obtained by solving the following optimization problem:


$$\begin{array}{c} h^{k}=\text{arg}{min}_{h\in H}E[\ell \left(y,F^{k-\text{1}}(Z)+h(Z)\right]\;\;, \end{array}$$

which ensures that each successive model corrects the residual errors of the previous approximation [52]. The distinctive feature of CatBoost compared to other boosting algorithms, such as XGBoost and LightGBM lies in its implementation of ordered boosting. Instead of computing categorical feature statistics using the entire dataset, which would introduce target leakage, CatBoost calculates target-based statistics in an ordered fashion: for each sample, the statistic is derived only from the preceding observations. This approach mitigates the issue of prediction shift and provides unbiased estimates of categorical encodings, leading to improved generalization [52–54]. After K iterations, the final predictive function can be expressed as:


$$\begin{array}{c} F(Z)=\;\sum_{k=\text{1}}^{K}\eta_{k}h^{k}(Z), \end{array}$$

yielding a strong ensemble model that integrates multiple weak learners while preserving robustness against overfitting. Consequently, CatBoost achieves high accuracy in tasks with heterogeneous and categorical data, while reducing the need for extensive data preprocessing.

#### 3.2.2 Multilayer Perceptron (MLP).

A Multilayer Perceptron (MLP) is a mathematical model used to estimate nonlinear relationships between variables, whether in classification or prediction tasks. This model consists of three main types of layers: an input layer, one or more hidden layers, and an output layer [55]. The internal structure of each neuron in this network relies on computing a linear combination of inputs, represented by the formula:


$$\begin{array}{c} M_{k}=\sum_{j=\text{1}}^{n}w_{kj}x_{j}+\theta_{k}, \end{array}$$

where $w_{kj}$ denotes the weights associated with the input variables $x_{j}$_j_, and $\theta_{k}$ is the bias term. The resulting value $M_{k}$ is then passed through a nonlinear activation function. In binary classification tasks, the Sigmoid function is commonly used, and is expressed as:


$$\begin{array}{c} A_{k}=\frac{\text{1}}{\text{1}+e^{-M_{k}}}, \end{array}$$

which transforms the output into a probability ranging between 0 and 1. Accordingly, the final output of the neuron is computed using


$$\begin{array}{c} Y_{k}=A_{k}(\sum_{j=\text{1}}^{n}w_{kj}x_{j}+\theta_{k}), \end{array}$$

enabling the model to predict classifications based on the generated probabilities. These equations demonstrate how data is processed inside an MLP in a sequential manner, allowing for the discovery of nonlinear patterns in classification data [55,56].

## 4. Performance assessment indicators

To evaluate the predictive capability of the proposed models, the dataset is partitioned such that 80% is used for training and the remaining 20% is reserved for testing. We utilized 5-fold cross-validation on the training dataset to ensure a robust evaluation and to reduce the risk of overfitting. The performance of machine learning (ML) classifiers is evaluated using a set of well-established metrics. These include accuracy (Acc), precision (Prec.), recall, F1-score, the Matthews Correlation Coefficient (MCC), and Cohen’s Kappa (κ). Each of these metrics provides a distinct perspective on the model’s behavior, particularly in addressing class imbalance or assessing agreement beyond chance levels. The metrics are calculated based on the confusion matrix (Table 2), which captures the relationships between actual labels and predicted outcomes. A detailed overview of these metrics, along with their mathematical formulations [51,57,58], is presented in Table 3.

**Table 2:** Confusion matrix.

	Actual: Positive	Actual: Negative
Predicted: Positive	True Positive (TP)	False Positive (FP)
Predicted: Negative	False Negative (FN)	True Negative (TN)

**Table 3:** Performance metrics formulas.

Metric	Formula	Value Range	Interpretation
Accuracy	$\frac{TP+TN}{TP+TN+FP+FN}$	0–1	Thresholds vary by context; values ≥ 0.70 are often acceptable, and ≥ 0.80 indicate strong performance [59,60].
Precision	$\frac{\text{TP}}{\text{TP}+\text{FP}}$	0–1	
Recall	$\frac{\text{TP}}{\text{TP}+\text{FN}}$	0–1	
F1-score	$\frac{\text{2}\times (\text{Precision}\times \text{Recall})}{\text{Precision}\times \text{Recall}}$	0–1	
Matthews Correlation Coefficient (MCC)	$\frac{\left(\text{TP}\times \text{TN})-(\text{FP}\times \text{FN}\right)}{\sqrt{\left(\text{TP}+\text{FP})(\text{TP}+\text{FN})(\text{TN}+\text{FP})(\text{TN}+\text{FN}\right)}}$	−1 to +1	MCC measures the correlation between predicted and actual class values. Values close to +1 indicate strong correlation, values near 0 indicate no correlation, and values close to −1 indicate inverse correlation [57].
Cohen’s Kappa (κ)	$\kappa =\frac{P_{\text{0}}-P_{e}}{\text{1}-P_{e}}$	−1 to +1	κ quantifies the level of agreement between predicted and actual classifications. A κ value of 1 indicates perfect agreement, while κ ≤ 0 suggests no agreement beyond random chance [61].
Receiver Operating Characteristic Curve (ROC)	The relationship between: $\text{TPR}=\frac{\text{TP}}{\text{TP}+\text{FN}}\;,\text{ FPR}=\frac{\text{FP}}{\text{FP}+\text{TN}}$	(0,0) to (1,1)	ROC curve illustrates the trade-off between the TPR and FPR across different classification thresholds. A curve that bows toward the top-left corner indicates better model performance. The diagonal line (TPR = FPR) represents random guessing [62].
AUC (Area Under the ROC Curve)	$AUC=\int_{\text{0}}^{\text{1}}TPR(FPR)dFPR$	0–1	AUC quantifies a model’s ability to distinguish between classes. Values closer to 1 indicate stronger discriminative performance, while AUC = 0.5 indicates no discriminative ability [51].

$*P_{\text{0}}$ is the observed agreement, $P_{e}$ is the expected agreement by chance, True Positive Rate (TPR), False Positive Rate (FPR).

## 5. Results

### 5.1 Key feature selection using Whale Optimization Algorithm (WOA) between the full (HbA1c ≥ 6.5%) and reduced (HbA1c > 8%) dataset

The proposed Whale Optimization Algorithm (WOA) is used to select the most relevant features in the predictive model, based on the preprocessed dataset. In this algorithm, each ‘whale’ represents a set of features, where a value of (1) indicates that the feature is selected, and a value of (0) indicates that it is not. Each whale’s feature set is treated as a potential solution to the feature selection problem. During each iteration, a fitness function is calculated for every whale using the RF model. This function measures the quality of the selected features based on their predictive power. After evaluating all whales, their positions are updated to move closer to the best solution found so far. This update process is based on three main behaviors inspired by the natural movement of whales: encircling, spiral movement, and exploration. These behaviors help strike a balance between exploiting known reasonable solutions and exploring new ones. As iterations progress, the search space gradually narrows, improving the chances of finding the optimal solution. If a whale’s new position yields better performance (based on the fitness score), it is adopted as the new optimal position. Otherwise, the previous position is retained. The algorithm continues to update positions across iterations, steadily improving performance, until it reaches the optimal set of features that ensures high accuracy while minimizing the number of variables used.

The algorithm was implemented with multiple settings to assess the effect of population size and iteration count on feature selection quality. The number of whales N was set to values {10, 20, 30, 40, 50, 60}, and the maximum number of iterations T was set to 100. An early stopping criterion was applied, whereby optimization terminated if no improvement in the best fitness value was observed for 10 consecutive iterations. The patience threshold of 10 iterations was empirically determined through preliminary experiments to balance computational efficiency with sufficient exploration of the search space, consistent with commonly adopted practices in metaheuristic optimization literature [48,49]. The fitness function was based on the classification accuracy of an RF classifier. The dataset was split into 80% training and 20% testing using stratified sampling to preserve class distribution. For the training data, 5-fold cross-validation was employed to ensure robust evaluation and prevent overfitting. The RF classifier was configured with 100 decision trees (n_estimators = 100), a maximum depth of 5 (max_depth = 5), and a fixed random seed (random_state = 42) for reproducibility. The final selection criterion preferred the solution with the highest accuracy, and in cases of equal accuracy, the subset with the fewest features was selected.

Fig 4 illustrates the effect of varying the number of whales and iterations on the optimization performance of the WOA across the two datasets. Each line represents the learning trajectory for a specific whale population size (N = 10, 20, 30, 40, 50, and 60), showing how the best fitness value evolves as the number of iterations increases. Each marker corresponds to the highest classification accuracy achieved by the whale population up to that iteration, thereby representing the best-so-far solution identified during the optimization process. In the full dataset (HbA1c ≥ 6.5%), model accuracy begins at approximately 0.73 and gradually improves as the number of iterations increases. During the early stages of the optimization process (T = 0–40), larger whale populations generally achieve higher fitness values than smaller populations, suggesting improved coverage of the feature search space and a greater likelihood of identifying high-quality feature subsets. As the optimization progresses, all curves exhibit a convergence pattern, with the highest accuracy reaching approximately 0.84 for N = 60. The gradual flattening of the curves after approximately 60–70 iterations indicates a transition from exploration to exploitation, where additional iterations provide only marginal improvements in fitness. In contrast, the reduced dataset (HbA1c > 8%) starts with a higher initial accuracy of approximately 0.81 and demonstrates a faster convergence rate throughout the optimization process. The best-performing configuration (N = 60) reaches an accuracy of approximately 0.89, exceeding the maximum accuracy obtained in the full dataset. Furthermore, the steeper increase in accuracy observed during the first 50–60 iterations suggests that the reduced dataset presents a simpler optimization landscape, enabling the algorithm to identify high-quality feature subsets more efficiently. The convergence curves also become stable earlier, at approximately T = 60–70 for the reduced dataset compared to T = 80–90 for the full dataset, indicating that fewer iterations are required to approach the optimal solution.

**Fig 4:**
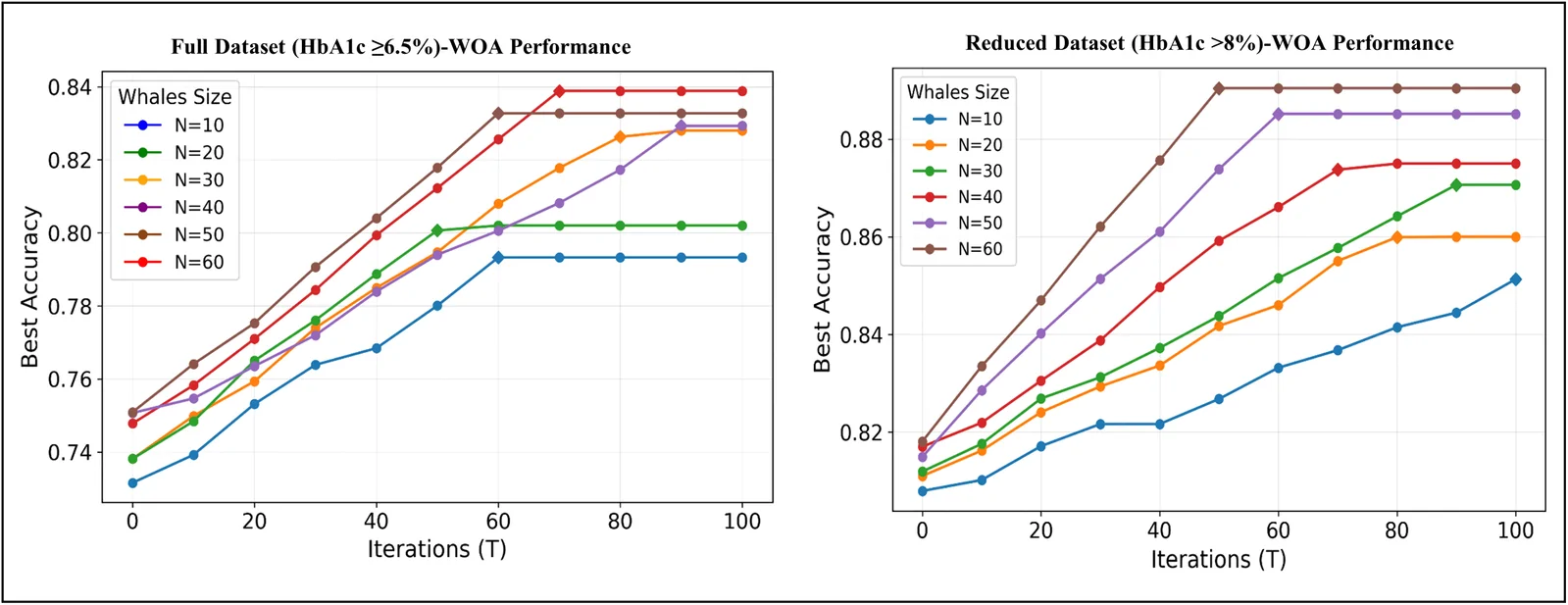
Impact of the number of whales and iterations on model accuracy using WOA on the full dataset (HbA1c ≥ 6.5%) and the reduced dataset (HbA1c > 8%).

Overall, Fig 4 demonstrates that increasing the whale population size generally improves optimization performance by enhancing the balance between exploration and exploitation. However, the performance gains become progressively smaller beyond N = 50–60, suggesting diminishing returns from further increases in population size. The plateau observed in the later iterations indicates that the algorithm has converged to a stable feature subset, supporting the adequacy of the selected optimization settings and confirming the robustness of the feature-selection process.

Fig 5 presents the optimal features identified by recording their selection frequency across different WOA configurations, which varied in the number of whales and iterations. Features with a selection frequency above 80% were considered stable and retained as optimal. To further validate this cut-off, a sensitivity analysis was conducted by retraining the RF classifier using feature subsets obtained at different stability thresholds (70%, 80%, and 90%). The results showed that model accuracy increased from 0.82 to 0.85 as the threshold rose from 70% to 80%, and then plateaued at 90%. This consistency demonstrates that the 80% threshold provides a robust and balanced trade-off between feature stability and predictive performance. In the full dataset (HbA1c ≥ 6.5%), 14 features out of 19 were retained, namely BMI, HbA1c, TG, type of food, age, SBP, HDL, vitamin-D, physical activity, marital status, occupation, gender, smoking, and TC. In contrast, in the reduced dataset (HbA1c > 8%), only 9 features out of 19 were selected, including HbA1c, HDL, type of food, age, BMI, TG, smoking, occupation and gender, reflecting the ability of WOA to reduce the number of variables while preserving the most influential predictors.

**Fig 5:**
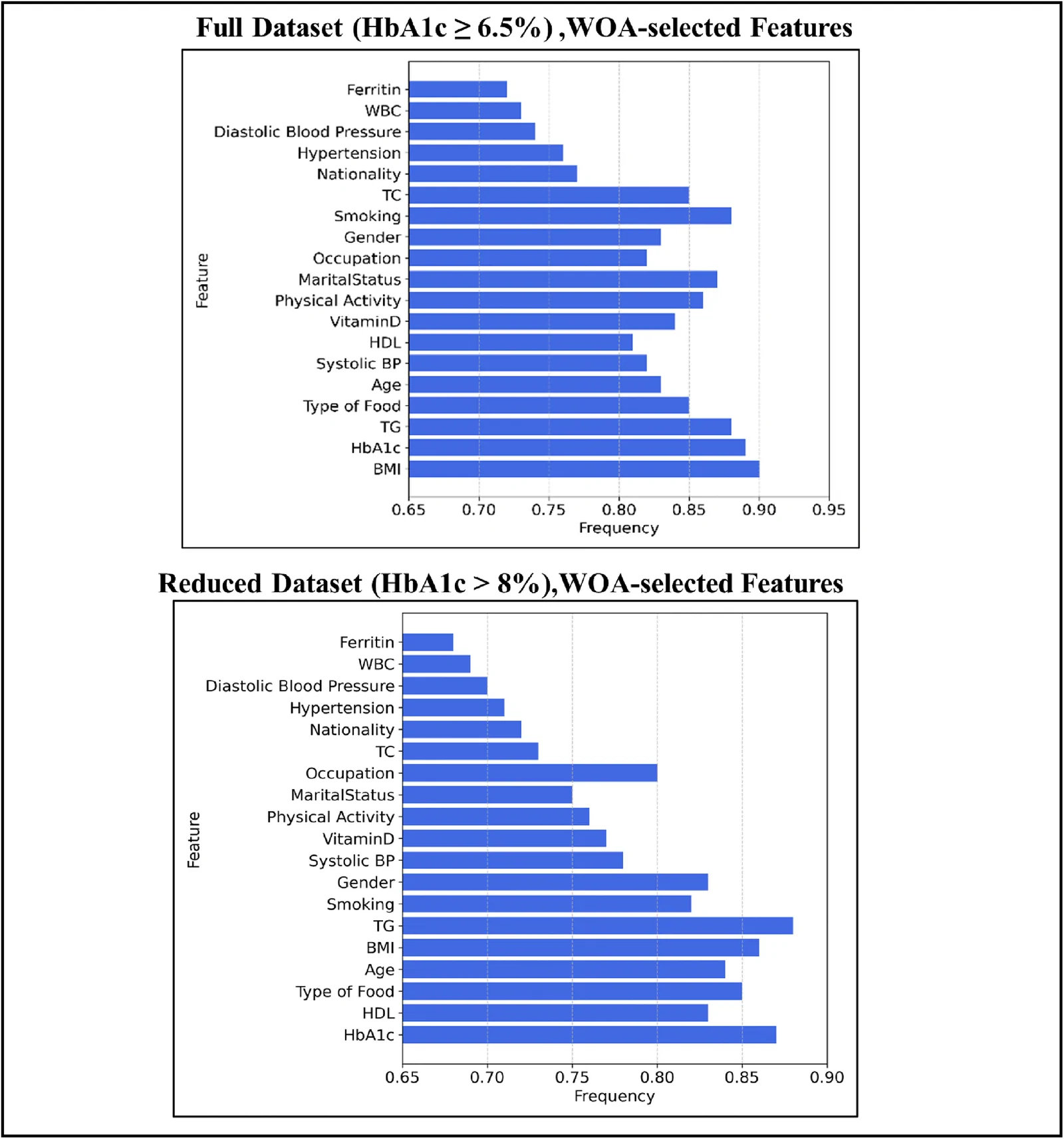
Feature selection frequencies using WOA in the full dataset (HbA1c ≥ 6.5%) and the reduced dataset (HbA1c > 8%).

### 5.2 Key feature selection using Black Widow Optimization (BWO) between the full (HbA1c ≥ 6.5%) and reduced (HbA1c > 8%) dataset

The Black Widow Optimization (BWO) algorithm, inspired by the mating, cannibalism, and survival mechanisms of black widow spiders, was employed in this study as a bio-inspired feature selection strategy. In this framework, each individual in the population represents a candidate solution encoded as a binary vector, where a value of 1 indicates the inclusion of a feature, and 0 its exclusion. The algorithm iteratively refines the population through three core operations: reproduction, cannibalism, and mutation, which aim to enhance the quality of selected feature subsets over generations. The fitness of each individual was evaluated based on the classification accuracy of an RF classifier, configured with 100 decision trees (n_estimators = 100), a maximum depth of 5 (max_depth = 5), and a fixed random seed (random_state = 42) to ensure reproducibility. At each iteration, the best-so-far accuracy defined as the highest 5-fold cross-validated RF classification accuracy achieved by any candidate feature subset up to and including that iteration was recorded to monitor convergence behaviour.

To ensure reproducibility, the BWO algorithm was implemented with a crossover rate of 0.7, a cannibalism rate of 0.4, and a mutation rate of 0.1, across population sizes N ∈ {10, 20, 30, 40, 50, 60} and a maximum of 100 iterations. Fitness evaluation was performed exclusively on the training set using 5-fold cross-validation, with the best-so-far accuracy updated at each iteration to ensure robust internal validation throughout the optimization process. An early stopping criterion was incorporated, whereby the optimization process was terminated if no improvement in the best fitness value was observed for 10 consecutive iterations. The patience threshold of 10 iterations was determined through preliminary experiments to balance computational efficiency with adequate exploration of the search space, consistent with empirical tuning practices commonly adopted in metaheuristic optimization studies [48,49]. For convergence visualization consistency, results were displayed across the full 100-iteration horizon.

Fig 6 presents the convergence behaviour of the BWO algorithm across varying population sizes, ranging from 10 to 60 spiders, applied to two datasets: the full dataset (HbA1c ≥ 6.5%) and the reduced high-risk dataset (HbA1c > 8%). In the full dataset, initial best-so-far accuracy values range from approximately 0.71 for smaller populations (10 spiders) to 0.79 for larger populations (60 spiders), reflecting the advantage of broader initial exploration through larger population sampling. Over 100 iterations, all population configurations exhibit a consistent upward trajectory, with terminal accuracy values ranging from approximately 0.76 (10 spiders) to 0.88 (60 spiders), indicating that larger populations maintain stronger exploration capability and are less susceptible to premature convergence.

**Fig 6:**
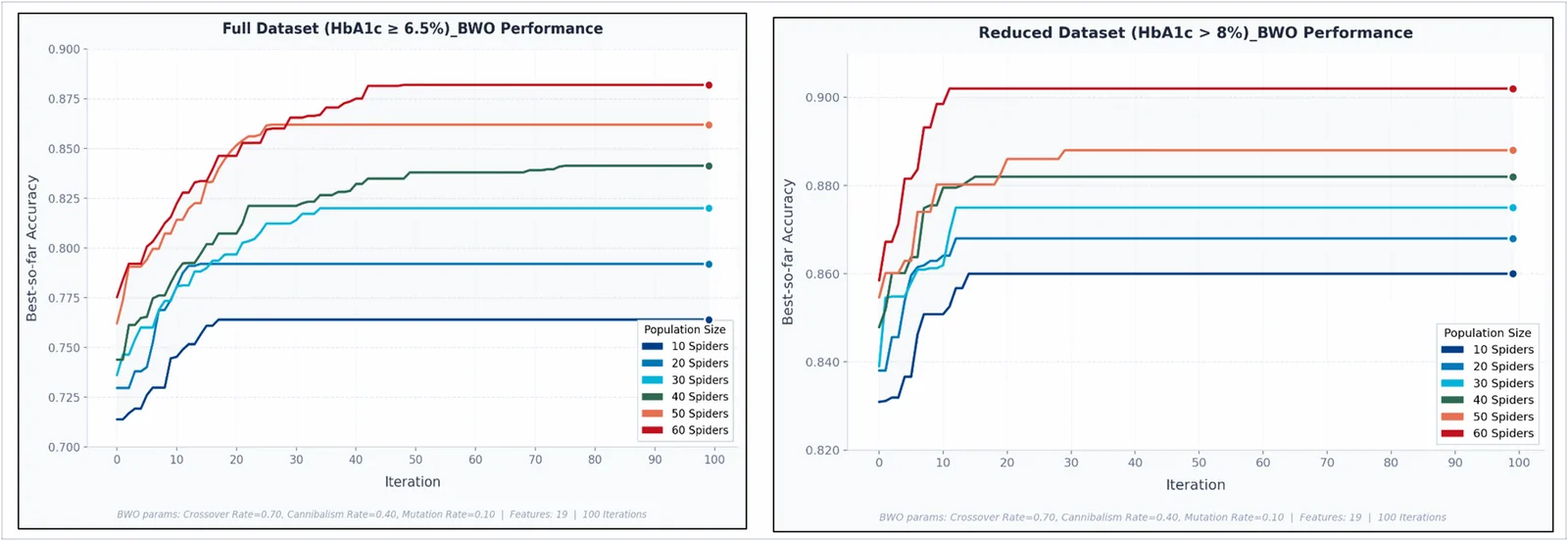
Impact of the number of spiders and iterations on model accuracy using BWO on the full dataset (HbA1c ≥ 6.5%) and the reduced dataset (HbA1c > 8%).

In contrast, the reduced dataset begins with notably higher initial accuracies, ranging from approximately 0.83 (10 spiders) to 0.85 (60 spiders) across all population sizes, and demonstrates faster and more compact convergence, stabilizing between approximately 0.86 (10 spiders) and 0.90 (60 spiders) by iteration 100, suggesting a comparatively less complex feature-search space within the high-risk subgroup. The visual separation among the curves further indicates that larger populations, particularly those comprising 50 and 60 spiders, consistently achieve higher and more stable accuracy. Most convergence curves plateau between approximately 10 and 30 iterations in the reduced dataset, and between 20 and 50 iterations in the full dataset, confirming that the algorithm reaches a stable convergence state well within the allocated 100-iteration horizon. Overall, these results demonstrate that increasing the spider population size enhances exploration capability, reduces performance variability, and contributes to more reliable convergence behaviour, with medium-to-large population sizes (N ≥ 40) consistently demonstrating superior performance for BWO-based feature selection in both diabetic prediction scenarios.

Fig 7 presents the results of the BWO algorithm in identifying key features associated with VI within the full dataset (HbA1c ≥ 6.5%). Features that appeared in more than 80% of the best solutions were considered important and therefore retained. As shown in Fig 7(A) and 7(B), 12 out of the original 19 features were selected, including HbA1c, HDL, physical activity, TG, BMI, smoking, type of food,SBP, TC, age, gender and occupation.

**Fig 7:**
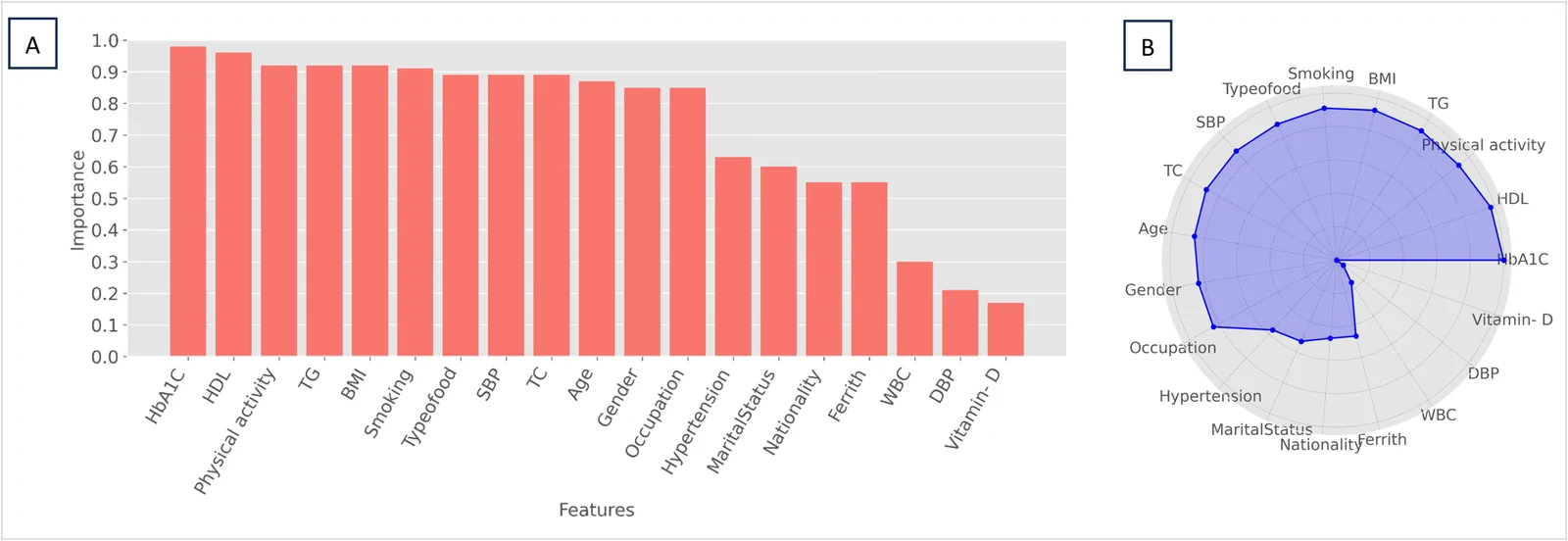
Feature importance from the BWO algorithm on the full dataset (HbA1c ≥ 6.5%). (A) Bar plot. (B) Radar plot.

In the reduced dataset (HbA1c > 8%), the BWO algorithm selected 10 important features as the most relevant predictors of VI based on a selection frequency greater than 80% across the best solutions, namely HbA1c, TG, occupation, age, smoking, type of food, BMI, hypertension, WBC, vitamin-D, as shown in Fig 8(A) and 8(B).

**Fig 8:**
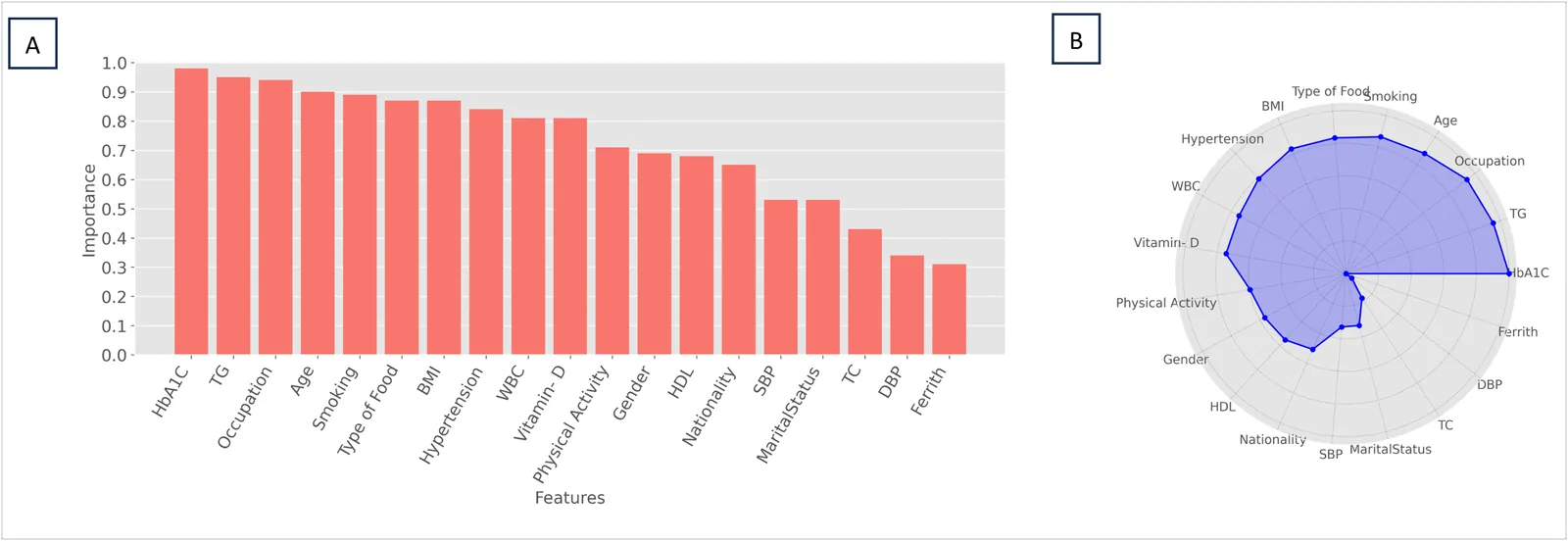
Feature importance from the BWO algorithm on the reduced dataset (HbA1c > 8%). (A) Bar plot. (B) Radar plot.

### 5.3 Model hyperparameter optimization

S2 Table presents the hyperparameter search grids and the final selected configurations for each of the 11 classification models utilized in this study. Hyperparameter tuning was performed separately for the full and reduced datasets and independently for all three approaches (Baseline, WOA, and BWO) using grid search with stratified 5-fold cross-validation within the training set only. Candidate hyperparameter combinations were evaluated using stratified 5-fold cross-validation, and the configuration achieving the highest mean cross-validated performance was selected. The final classifier was then refitted on the complete training set using the selected hyperparameters and evaluated on the independent test set.

### 5.4 Performance assessment of baseline machine learning models without feature selection in predicting visual impairment

This section evaluates the predictive performance of 11 machine learning models using the full set of 19 predictor variables to predict VI among patients with T2D across both the full dataset (HbA1c ≥ 6.5%) and the reduced high-risk dataset (HbA1c > 8%). Table 4 presents the predictive performance of the 11 classification models across the two datasets. As shown in Table 4, the MLP achieved the strongest performance among all classifiers in the full dataset (HbA1c ≥ 6.5%), with a testing accuracy of 0.88, precision of 0.89, recall of 0.89, F1-score of 0.89, MCC of 0.78, Kappa of 0.76, and AUC of 0.90. These findings indicate strong and balanced classification performance. Other models, such as XGBoost, CatBoost, and RF, also demonstrated competitive predictive performance, with testing accuracies of 0.87, 0.86, and 0.85, respectively, but performed slightly below the MLP across the evaluated metrics. In the reduced high-risk dataset (HbA1c > 8%), most classifiers demonstrated improved predictive performance compared with the full dataset. Among these, the MLP again achieved the highest overall performance, with a testing accuracy of 0.91, precision of 0.92, recall of 0.93, F1-score of 0.92, MCC of 0.88, Kappa of 0.86, and AUC of 0.94. This improvement suggests that focusing on a more clinically relevant high-risk subgroup enhances predictive discrimination. Overall, the superior performance of the MLP across both datasets, particularly in the reduced high-risk subgroup, supports the potential value of HbA1c-based patient stratification for improving VI prediction in T2D populations.

**Table 4:** Performance of baseline classification models without feature selection on full (HbA1c ≥ 6.5%) and reduced (HbA1c > 8%) datasets, with number of variables = 19.

			Models										
			Ada-Boost	LR	RF	DT	KNN	MLP	NB	SVM	XGBoost	CatBoost	GraBoost
**Training**	**Acc.**	**Full Model (HbA1c ≥ 6.5%)**	0.83 (0.80–0.86)	0.85 (0.83–0.87)	0.87 (0.85–0.89)	0.81 (0.78–0.84)	0.86 (0.84–0.88)	0.91 (0.89–0.93)	0.83 (0.80–0.86)	0.86 (0.84–0.88)	0.89 (0.87–0.91)	0.88 (0.86–0.90)	0.84 (0.81–0.87)
	**Prec.**		0.84 (0.81–0.87)	0.84 (0.81–0.87)	0.87 (0.85–0.89)	0.81 (0.78–0.84)	0.87 (0.85–0.89)	0.92 (0.90–0.94)	0.83 (0.80–0.86)	0.85 (0.83–0.87)	0.90 (0.88–0.92)	0.89 (0.87–0.91)	0.85 (0.83–0.87)
	**Recall**		0.83 (0.80–0.86)	0.83 (0.80–0.86)	0.86 (0.84–0.88)	0.80 (0.77–0.83)	0.88 (0.86–0.90)	0.90 (0.88–0.92)	0.83 (0.80–0.86)	0.87 (0.85–0.89)	0.89 (0.87–0.91)	0.88 (0.86–0.90)	0.84 (0.81–0.87)
	**F1-Score**		0.83 (0.80–0.86)	0.85 (0.80–0.86)	0.86 (0.84–0.88)	0.80 (0.77–0.83)	0.87 (0.85–0.89)	0.91 (0.89–0.93)	0.83 (0.80–0.86)	0.86 (0.84–0.88)	0.89 (0.87–0.91)	0.88 (0.86–0.90)	0.84 (0.81–0.87)
	**MCC**		0.68 (0.65–0.71)	0.68 (0.65–0.71)	0.76 (0.73–0.79)	0.64 (0.61–0.67)	0.74 (0.71–0.77)	0.82 (0.79–0.85)	0.68 (0.65–0.71)	0.74 (0.71–0.77)	0.80 (0.77–0.83)	0.78 (0.75–0.81)	0.70 (0.67–0.73)
	**K**		0.67 (0.64–0.70)	0.67 (0.64–0.70)	0.75 (0.72–0.78)	0.62 (0.59–0.65)	0.73 (0.70–0.76)	0.80 (0.77–0.83)	0.66 (0.63–0.69)	0.73 (0.70–0.76)	0.79 (0.76–0.82)	0.77 (0.74–0.80)	0.69 (0.66–0.72)
	**AUC**		0.85 (0.80–0.90)	0.87 (0.85–0.89)	0.89 (0.87–0.91)	0.89 (0.87–0.91)	0.83 (0.80–0.86)	0.92 (0.90–0.94)	0.93 (0.91–0.95)	0.88 (0.86–0.90)	0.89 (0.87–0.91)	0.91 (0.89–0.93)	0.90 (0.88–0.92)
**Testing**	**Acc.**	**Full Model (HbA1c ≥ 6.5%)**	0.81 (0.76–0.86)	0.83 (0.78–0.88)	0.85 (0.80–0.90)	0.78 (0.72–0.84)	0.84 (0.79–0.89)	0.88 (0.83–0.93)	0.82 (0.77–0.87)	0.84 (0.79–0.89)	0.87 (0.82–0.92)	0.86 (0.81–0.91)	0.82 (0.77–0.87)
	**Prec.**		0.82 (0.77–0.87)	0.82 (0.77–0.87)	0.85 (0.80–0.90)	0.78 (0.72–0.84)	0.85 (0.80–0.90)	0.89 (0.85–0.93)	0.82 (0.77–0.87)	0.83 (0.78–0.88)	0.88 (0.83–0.93)	0.87 (0.82–0.92)	0.83 (0.78–0.88)
	**Recall**		0.81 (0.76–0.86)	0.83 (0.78–0.88)	0.85 (0.80–0.90)	0.78 (0.72–0.84)	0.84 (0.79–0.89)	0.89 (0.85–0.93)	0.82 (0.77–0.87)	0.84 (0.79–0.89)	0.88 (0.83–0.93)	0.86 (0.81–0.91)	0.83 (0.78–0.88)
	**F1-Score**		0.81 (0.76–0.86)	0.83 (0.78–0.88)	0.85 (0.80–0.90)	0.78 (0.72–0.84)	0.84 (0.79–0.89)	0.89 (0.85–0.93)	0.82 (0.77–0.87)	0.84 (0.79–0.89)	0.88 (0.83–0.93)	0.86 (0.81–0.91)	0.83 (0.78–0.88)
	**MCC**		0.66 (0.59–0.73)	0.70 (0.64–0.76)	0.74 (0.68–0.80)	0.61 (0.54–0.68)	0.71 (0.65–0.77)	0.78 (0.72–0.84)	0.67 (0.60–0.74)	0.71 (0.65–0.77)	0.76 (0.70–0.82)	0.74 (0.68–0.80)	0.68 (0.62–0.74)
	**K**		0.65 (0.58–0.72)	0.69 (0.63–0.75)	0.73 (0.67–0.79)	0.60 (0.53–0.67)	0.70 (0.64–0.76)	0.76 (0.70–0.82)	0.66 (0.59–0.73)	0.70 (0.64–0.76)	0.75 (0.69–0.81)	0.73 (0.67–0.79)	0.67 (0.60–0.74)
	**AUC**		0.84 (0.79–0.89)	0.86 (0.81–0.91)	0.86 (0.81–0.91)	0.80 (0.74–0.86)	0.89 (0.84–0.94)	0.90 (0.86–0.94)	0.85 (0.80–0.90)	0.86 (0.81–0.91)	0.88 (0.83–0.93)	0.87 (0.82–0.92)	0.85 (0.80–0.90)
**Training**	**Acc.**	**Reduced Model (HbA1c > 8%)**	0.86 (0.84–0.88)	0.86 (0.84–0.88)	0.91 (0.89–0.93)	0.84 (0.81–0.87)	0.87 (0.85–0.89)	0.95 (0.93–0.97)	0.88 (0.86–0.90)	0.85 (0.83–0.87)	0.92 (0.90–0.94)	0.91 (0.89–0.93)	0.90 (0.88–0.92)
	**Prec.**		0.86 (0.84–0.88)	0.86 (0.84–0.88)	0.91 (0.89–0.93)	0.84 (0.81–0.87)	0.87 (0.85–0.89)	0.96 (0.95–0.97)	0.88 (0.86–0.90)	0.85 (0.83–0.87)	0.93 (0.91–0.95)	0.92 (0.90–0.94)	0.91 (0.89–0.93)
	**Recall**		0.85 (0.83–0.87)	0.86 (0.84–0.88)	0.90 (0.88–0.92)	0.83 (0.80–0.86)	0.87 (0.85–0.89)	0.94 (0.92–0.96)	0.87 (0.85–0.89)	0.85 (0.83–0.87)	0.92 (0.90–0.94)	0.91 (0.89–0.93)	0.89 (0.87–0.91)
	**F1-Score**		0.85 (0.83–0.87)	0.86 (0.84–0.88)	0.90 (0.88–0.92)	0.83 (0.80–0.86)	0.87 (0.85–0.89)	0.95 (0.93–0.97)	0.87 (0.85–0.89)	0.85 (0.83–0.87)	0.93 (0.91–0.95)	0.91 (0.89–0.93)	0.90 (0.88–0.92)
	**MCC**		0.75 (0.72–0.78)	0.76 (0.73–0.79)	0.84 (0.81–0.87)	0.70 (0.67–0.73)	0.78 (0.75–0.81)	0.90 (0.88–0.92)	0.80 (0.77–0.83)	0.74 (0.71–0.77)	0.86 (0.84–0.88)	0.85 (0.83–0.87)	0.83 (0.80–0.86)
	**K**		0.73 (0.70–0.76)	0.74 (0.71–0.77)	0.83 (0.80–0.86)	0.68 (0.65–0.71)	0.76 (0.73–0.79)	0.88 (0.86–0.90)	0.78 (0.75–0.81)	0.72 (0.69–0.75)	0.85 (0.83–0.87)	0.84 (0.81–0.87)	0.82 (0.79–0.85)
	**AUC**		0.90 (0.88–0.92)	0.90 (0.88–0.92)	0.93 (0.91–0.95)	0.83 (0.80–0.86)	0.90 (0.88–0.92)	0.97 (0.96–0.98)	0.91 (0.89–0.93)	0.89 (0.87–0.91)	0.95 (0.93–0.97)	0.95 (0.93–0.97)	0.92 (0.90–0.94)
**Testing**	**Acc.**	**Reduced Model (HbA1c > 8%)**	0.85 (0.80–0.90)	0.85 (0.80–0.90)	0.88 (0.83–0.93)	0.81 (0.76–0.86)	0.86 (0.81–0.91)	**0.91** (0.87–0.95)	0.86 (0.81–0.91)	0.84 (0.79–0.89)	0.89 (0.85–0.93)	0.89 (0.85–0.93)	0.87 (0.82–0.92)
	**Prec.**		0.84 (0.79–0.89)	0.85 (0.80–0.90)	0.88 (0.83–0.93)	0.80 (0.74–0.86)	0.86 (0.81–0.91)	0.92 (0.88–0.96)	0.86 (0.81–0.91)	0.83 (0.78–0.88)	0.90 (0.86–0.94)	0.90 (0.86–0.94)	0.88 (0.83–0.93)
	**Recall**		0.85 (0.80–0.90)	0.85 (0.80–0.90)	0.89 (0.85–0.93)	0.79 (0.73–0.85)	0.85 (0.80–0.90)	0.93 (0.89–0.97)	0.86 (0.81–0.91)	0.84 (0.79–0.89)	0.91 (0.87–0.95)	0.91 (0.87–0.95)	0.87 (0.82–0.92)
	**F1-Score**		0.84 (0.79–0.89)	0.85 (0.80–0.90)	0.88 (0.83–0.93)	0.79 (0.73–0.85)	0.85 (0.80–0.90)	0.92 (0.88–0.96)	0.86 (0.81–0.91)	0.83 (0.78–0.88)	0.90 (0.86–0.94)	0.90 (0.86–0.94)	0.87 (0.82–0.92)
	**MCC**		0.75 (0.69–0.81)	0.76 (0.70–0.82)	0.82 (0.77–0.87)	0.68 (0.62–0.74)	0.77 (0.71–0.83)	0.88 (0.83–0.93)	0.78 (0.72–0.84)	0.74 (0.68–0.80)	0.84 (0.79–0.89)	0.84 (0.79–0.89)	0.81 (0.76–0.86)
	**K**		0.72 (0.66–0.78)	0.73 (0.67–0.79)	0.80 (0.74–0.86)	0.66 (0.59–0.73)	0.74 (0.68–0.80)	0.86 (0.81–0.91)	0.76 (0.70–0.82)	0.71 (0.65–0.77)	0.83 (0.78–0.88)	0.83 (0.78–0.88)	0.79 (0.73–0.85)
	**AUC**		0.87 (0.82–0.92)	0.87 (0.82–0.92)	0.90 (0.86–0.94)	0.80 (0.74–0.86)	0.87 (0.82–0.92)	0.94 (0.91–0.97)	0.88 (0.83–0.93)	0.86 (0.81–0.91)	0.92 (0.88–0.96)	0.92 (0.88–0.96)	0.89 (0.84–0.94)

Fig 9 illustrates the AUC scores with corresponding 95% confidence intervals for all classifiers across both the full and reduced datasets. In the full dataset (HbA1c ≥ 6.5%), the MLP achieved the highest AUC of 0.90 (95% CI: 0.86–0.94), closely followed by KNN (AUC = 0.89, 95% CI: 0.84–0.94), XGBoost (AUC = 0.88, 95% CI: 0.83–0.93), and CatBoost (AUC = 0.87, 95% CI: 0.82–0.92). In the reduced high-risk dataset (HbA1c > 8%), the MLP achieved the highest AUC of 0.94 (95% CI: 0.91–0.97), followed by XGBoost and CatBoost, each with an AUC of 0.92 (95% CI: 0.88–0.96), and RF with an AUC of 0.90 (95% CI: 0.86–0.94). Overall, the MLP demonstrated the strongest discriminative performance across both datasets, particularly in the reduced high-risk subgroup, supporting its potential utility for VI risk stratification among patients with T2D.

**Fig 9:**
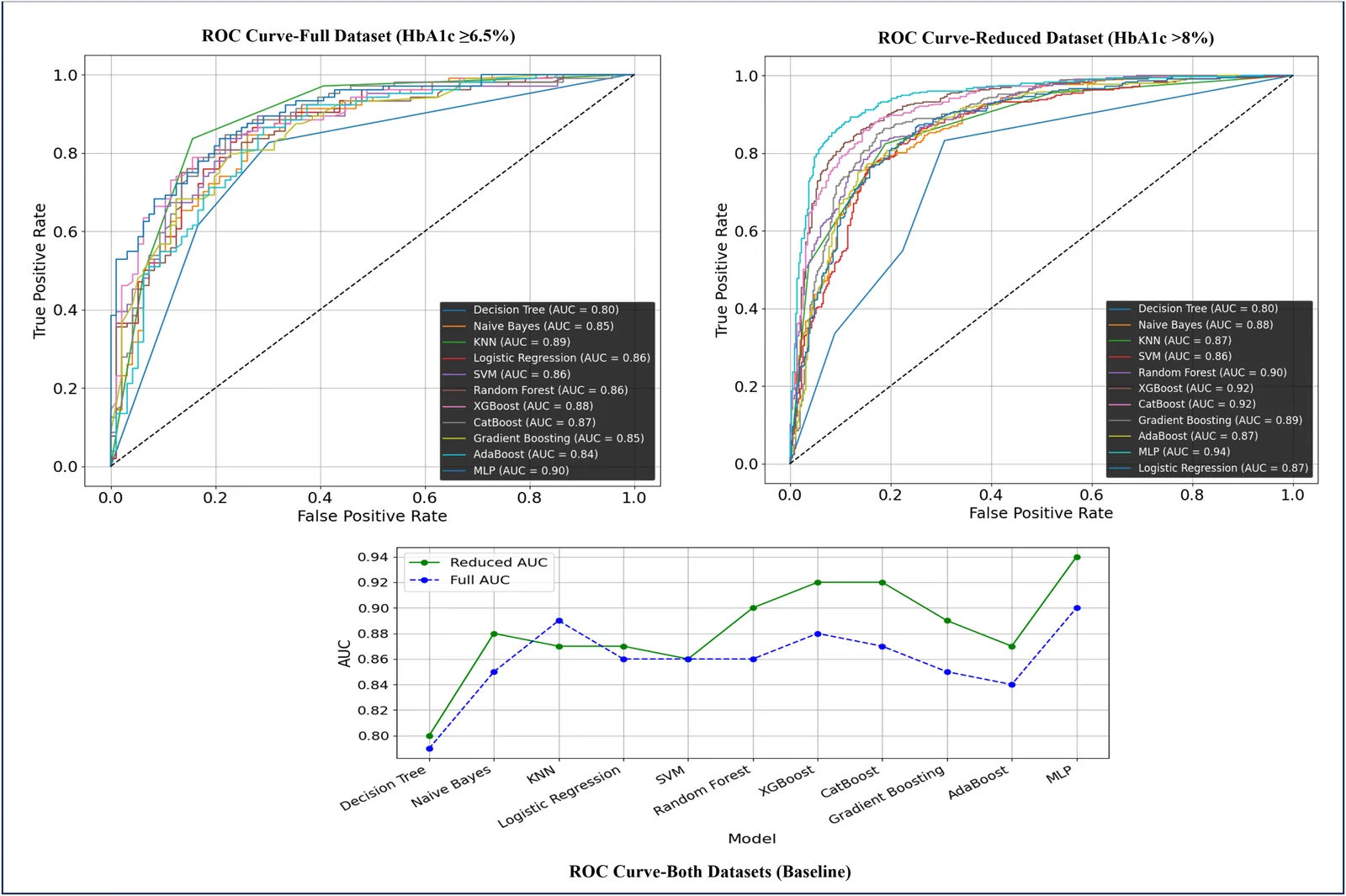
ROC curves comparing baseline classification models using full (HbA1c ≥ 6.5%) and reduced (HbA1c > 8%) datasets.

Fig 10 shows that the significant features identified by the MLP model in the reduced dataset (HbA1c > 8%) included 13 key variables out of the original 19. The feature importance ranking revealed that HbA1c was the most important predictor, followed by TG, age, smoking, type of food, BMI, HDL, gender, hypertension, occupation, vitamin D, physical activity, and WBC. Focusing on the high-risk subgroup with HbA1c > 8% enhanced predictive accuracy and improved model interpretability by narrowing the input to features most strongly associated with the risk of VI in patients with T2D.

**Fig 10:**
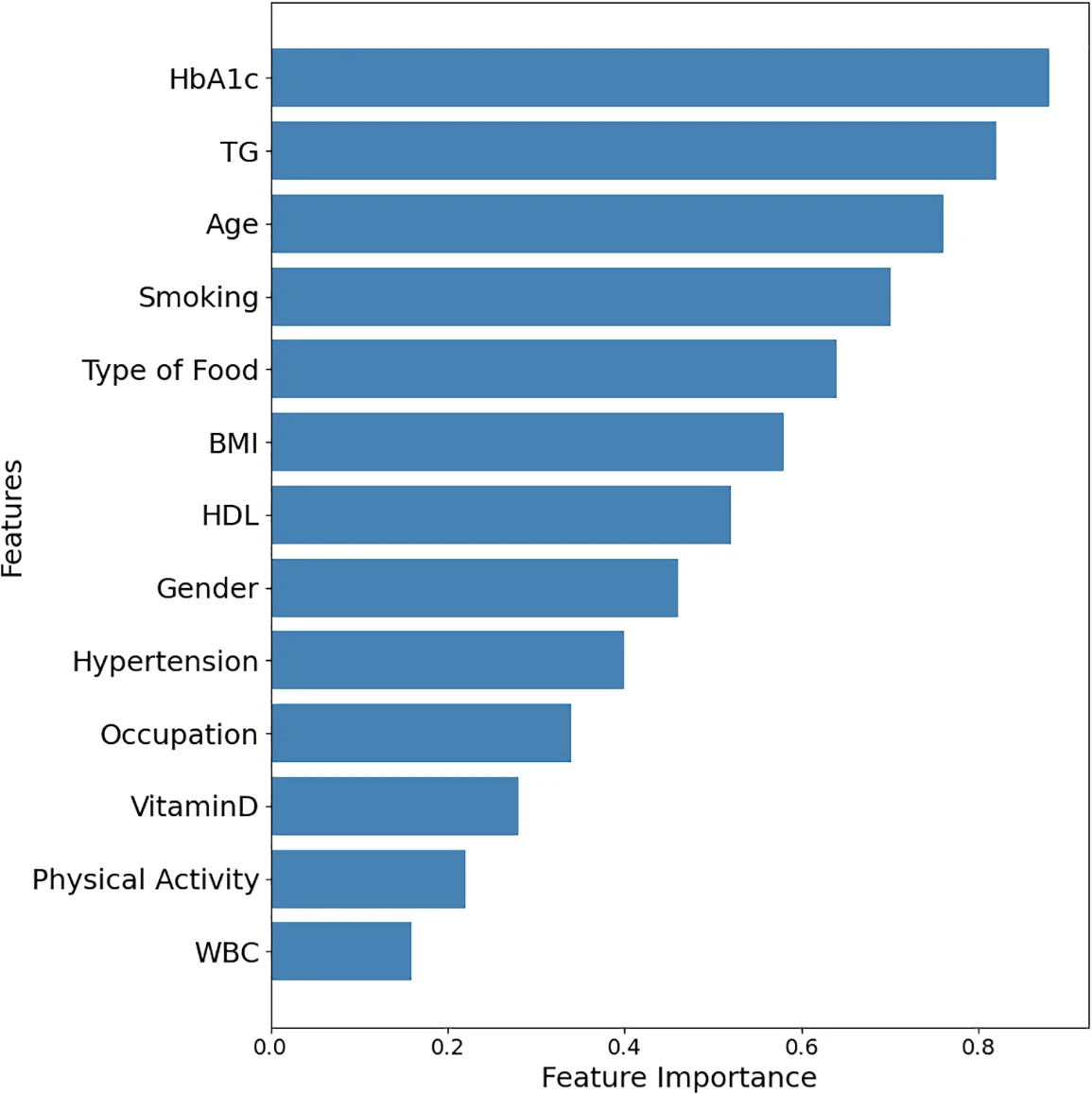
Key features ranked by the MLP model in the reduced dataset (HbA1c > 8%).

### 5.5 Performance assessment of machine learning models with Whale Optimization Algorithm (WOA) based feature selection in predicting visual impairment

The Whale Optimization Algorithm (WOA) was applied to both datasets the full dataset (HbA1c ≥ 6.5%) and the reduced dataset (HbA1c > 8%) to identify the optimal feature subset, as illustrated in Fig 4. Based on the selected features, Table 5 presents the performance of 11 machine learning classifiers trained on these optimized feature sets. In the full dataset, 14 features were selected. Among the evaluated classifiers, several models demonstrated strong predictive performance. The results show that XGBoost achieved the highest performance among all classifiers in this group, with a testing accuracy of 0.92, precision and recall of 0.92 and 0.93 respectively, an F1-score of 0.92, an MCC of 0.88, a kappa of 0.87, and AUC of 0.94. CatBoost followed closely, achieving an accuracy of 0.91, with precision, recall, and F1-score all at 0.91. Likewise, both the MLP and GraBoost models demonstrated competitive performance, each achieving a testing accuracy of 0.89. The MLP reported a precision of 0.88, a recall of 0.89, an F1-score of 0.88, an MCC of 0.83, a Kappa of 0.82, and AUC of 0.89, while GraBoost achieved a precision and recall of 0.89 and 0.88, respectively, an F1-score of 0.88, an MCC of 0.83, a Kappa of 0.82, and AUC of 0.90.

**Table 5:** Classification models with WOA-based feature selection for the full dataset (HbA1c ≥ 6.5%, number of variables = 14) and the reduced dataset (HbA1c > 8%, number of variables = 9).

			Models										
			Ada-Boost	LR	RF	DT	KNN	MLP	NB	SVM	XGBoost	CatBoost	GraBoost
**Training**	**Acc.**	**Full Model (HbA1c ≥ 6.5%)**	0.86 (0.84–0.88)	0.87 (0.85–0.89)	0.93 (0.91–0.95)	0.84 (0.81–0.87)	0.90 (0.88–0.92)	0.91 (0.89–0.93)	0.88 (0.86–0.90)	0.92 (0.90–0.94)	0.95 (0.93–0.97)	0.94 (0.92–0.96)	0.92 (0.90–0.94)
	**Prec.**		0.86 (0.84–0.88)	0.87 (0.85–0.89)	0.93 (0.91–0.95)	0.83 (0.80–0.86)	0.90 (0.88–0.92)	0.91 (0.89–0.93)	0.88 (0.86–0.90)	0.92 (0.90–0.94)	0.95 (0.93–0.97)	0.94 (0.92–0.96)	0.92 (0.90–0.94)
	**Recall**		0.85 (0.83–0.87)	0.86 (0.84–0.88)	0.92 (0.90–0.94)	0.82 (0.79–0.85)	0.89 (0.87–0.91)	0.90 (0.88–0.92)	0.87 (0.85–0.89)	0.91 (0.89–0.93)	0.94 (0.92–0.96)	0.93 (0.91–0.95)	0.91 (0.89–0.93)
	**F1-Score**		0.85 (0.83–0.87)	0.87 (0.85–0.89)	0.93 (0.91–0.95)	0.82 (0.79–0.85)	0.90 (0.88–0.92)	0.91 (0.89–0.93)	0.87 (0.85–0.89)	0.92 (0.90–0.94)	0.95 (0.93–0.97)	0.94 (0.92–0.96)	0.91 (0.89–0.93)
	**MCC**		0.76 (0.73–0.79)	0.78 (0.75–0.81)	0.89 (0.87–0.91)	0.70 (0.67–0.73)	0.84 (0.81–0.87)	0.86 (0.84–0.88)	0.80 (0.77–0.83)	0.88 (0.86–0.90)	0.91 (0.89–0.93)	0.90 (0.88–0.92)	0.88 (0.86–0.90)
	**K**		0.75 (0.72–0.78)	0.77 (0.74–0.80)	0.88 (0.86–0.90)	0.69 (0.66–0.72)	0.83 (0.80–0.86)	0.85 (0.83–0.87)	0.79 (0.76–0.82)	0.87 (0.85–0.89)	0.90 (0.88–0.92)	0.89 (0.87–0.91)	0.87 (0.85–0.89)
	**AUC**		0.91 (0.89–0.93)	0.92 (0.90–0.94)	0.96 (0.95–0.97)	0.87 (0.85–0.89)	0.94 (0.92–0.96)	0.95 (0.93–0.97)	0.92 (0.90–0.94)	0.96 (0.95–0.97)	0.97 (0.96–0.98)	0.97 (0.96–0.98)	0.95 (0.93–0.97)
**Testing**	**Acc.**	**Full Model** **(HbA1c ≥ 6.5%)**	0.85 (0.80–0.90)	0.86 (0.81–0.91)	0.90 (0.86–0.94)	0.80 (0.74–0.86)	0.88 (0.83–0.93)	0.89 (0.85–0.93)	0.87 (0.82–0.92)	0.90 (0.86–0.94)	0.92 (0.88–0.96)	0.91 (0.87–0.95)	0.89 (0.85–0.93)
	**Prec.**		0.84 (0.79–0.89)	0.85 (0.80–0.90)	0.90 (0.86–0.94)	0.79 (0.73–0.85)	0.88 (0.83–0.93)	0.88 (0.83–0.93)	0.86 (0.81–0.91)	0.89 (0.85–0.93)	0.92 (0.88–0.96)	0.91 (0.87–0.95)	0.89 (0.85–0.93)
	**Recall**		0.83 (0.78–0.88)	0.85 (0.80–0.90)	0.89 (0.85–0.93)	0.78 (0.72–0.84)	0.87 (0.82–0.92)	0.89 (0.85–0.93)	0.86 (0.81–0.91)	0.90 (0.86–0.94)	0.93 (0.89–0.97)	0.91 (0.87–0.95)	0.88 (0.83–0.93)
	**F1-Score**		0.83 (0.78–0.88)	0.85 (0.80–0.90)	0.89 (0.85–0.93)	0.78 (0.72–0.84)	0.87 (0.82–0.92)	0.88 (0.83–0.93)	0.86 (0.81–0.91)	0.89 (0.85–0.93)	0.92 (0.88–0.96)	0.91 (0.87–0.95)	0.88 (0.83–0.93)
	**MCC**		0.74 (0.68–0.80)	0.76 (0.70–0.82)	0.85 (0.80–0.90)	0.68 (0.62–0.74)	0.81 (0.76–0.86)	0.83 (0.78–0.88)	0.79 (0.73–0.85)	0.85 (0.80–0.90)	0.88 (0.83–0.93)	0.87 (0.82–0.92)	0.83 (0.78–0.88)
	**K**		0.73 (0.67–0.79)	0.75 (0.69–0.81)	0.84 (0.79–0.89)	0.67 (0.60–0.74)	0.80 (0.74–0.86)	0.82 (0.77–0.87)	0.78 (0.72–0.84)	0.84 (0.79–0.89)	0.87 (0.82–0.92)	0.86 (0.81–0.91)	0.82 (0.77–0.87)
	**AUC**		0.84 (0.79–0.89)	0.85 (0.80–0.90)	0.90 (0.86–0.94)	0.77 (0.71–0.83)	0.88 (0.83–0.93)	0.89 (0.84–0.94)	0.86 (0.81–0.91)	0.92 (0.88–0.96)	0.94 (0.91–0.97)	0.92 (0.88–0.96)	0.90 (0.86–0.94)
**Training**	**Acc.**	**Reduced Model (HbA1c > 8%)**	0.88 (0.86–0.90)	0.90 (0.88–0.92)	0.95 (0.93–0.97)	0.86 (0.84–0.88)	0.93 (0.91–0.95)	0.95 (0.93–0.97)	0.89 (0.87–0.91)	0.95 (0.93–0.97)	0.94 (0.92–0.96)	0.96 (0.95–0.97)	0.92 (0.90–0.94)
	**Prec.**		0.88 (0.86–0.90)	0.90 (0.88–0.92)	0.95 (0.93–0.97)	0.85 (0.83–0.87)	0.93 (0.91–0.95)	0.95 (0.93–0.97)	0.89 (0.87–0.91)	0.95 (0.93–0.97)	0.94 (0.92–0.96)	0.96 (0.95–0.97)	0.92 (0.90–0.94)
	**Recall**		0.87 (0.85–0.89)	0.89 (0.87–0.91)	0.94 (0.92–0.96)	0.84 (0.81–0.87)	0.92 (0.90–0.94)	0.94 (0.92–0.96)	0.88 (0.86–0.90)	0.94 (0.92–0.96)	0.94 (0.92–0.96)	0.96 (0.95–0.97)	0.91 (0.89–0.93)
	**F1-Score**		0.87 (0.85–0.89)	0.89 (0.87–0.91)	0.94 (0.92–0.96)	0.84 (0.81–0.87)	0.92 (0.90–0.94)	0.95 (0.93–0.97)	0.88 (0.86–0.90)	0.94 (0.92–0.96)	0.94 (0.92–0.96)	0.96 (0.95–0.97)	0.92 (0.90–0.94)
	**MCC**		0.80 (0.77–0.83)	0.84 (0.81–0.87)	0.92 (0.90–0.94)	0.75 (0.72–0.78)	0.89 (0.87–0.91)	0.92 (0.90–0.94)	0.82 (0.79–0.85)	0.91 (0.89–0.93)	0.91 (0.89–0.93)	0.93 (0.91–0.95)	0.87 (0.85–0.89)
	**K**		0.79 (0.76–0.82)	0.83 (0.80–0.86)	0.91 (0.89–0.93)	0.74 (0.71–0.77)	0.88 (0.86–0.90)	0.91 (0.89–0.93)	0.81 (0.78–0.84)	0.90 (0.88–0.92)	0.90 (0.88–0.92)	0.92 (0.90–0.94)	0.86 (0.84–0.88)
	**AUC**		0.90 (0.88–0.92)	0.91 (0.89–0.93)	0.95 (0.93–0.97)	0.84 (0.81–0.87)	0.93 (0.91–0.95)	0.96 (0.95–0.97)	0.90 (0.88–0.92)	0.95 (0.93–0.97)	0.95 (0.93–0.97)	0.98 (0.96–0.99)	0.93 (0.91–0.95)
**Testing**	**Acc.**	**Reduced Model (HbA1c > 8%)**	0.87 (0.82–0.92)	0.88 (0.83–0.93)	0.94 (0.91–0.97)	0.83 (0.78–0.88)	0.92 (0.88–0.96)	0.94 (0.91–0.97)	0.87 (0.82–0.92)	0.94 (0.91–0.97)	0.93 (0.89–0.97)	**0.95** (0.92–0.98)	0.91 (0.87–0.95)
	**Prec.**		0.86 (0.81–0.91)	0.88 (0.83–0.93)	0.93 (0.89–0.97)	0.82 (0.77–0.87)	0.92 (0.88–0.96)	0.94 (0.91–0.97)	0.87 (0.82–0.92)	0.93 (0.89–0.97)	0.93 (0.89–0.97)	0.95 (0.92–0.98)	0.91 (0.87–0.95)
	**Recall**		0.86 (0.81–0.91)	0.87 (0.82–0.92)	0.94 (0.91–0.97)	0.81 (0.76–0.86)	0.91 (0.87–0.95)	0.94 (0.91–0.97)	0.87 (0.82–0.92)	0.94 (0.91–0.97)	0.91 (0.89–0.97)	0.95 (0.92–0.98)	0.90 (0.86–0.94)
	**F1-Score**		0.86 (0.81–0.91)	0.87 (0.82–0.92)	0.94 (0.91–0.97)	0.81 (0.76–0.86)	0.91 (0.87–0.95)	0.94 (0.91–0.97)	0.87 (0.82–0.92)	0.93 (0.89–0.97)	0.93 (0.89–0.97)	0.95 (0.92–0.98)	0.90 (0.86–0.94)
	**MCC**		0.78 (0.72–0.84)	0.80 (0.74–0.86)	0.91 (0.87–0.95)	0.72 (0.66–0.78)	0.87 (0.82–0.92)	0.91 (0.87–0.95)	0.78 (0.72–0.84)	0.90 (0.86–0.94)	0.89 (0.85–0.93)	0.92 (0.88–0.96)	0.85 (0.80–0.90)
	**K**		0.77 (0.71–0.83)	0.79 (0.73–0.85)	0.90 (0.86–0.94)	0.71 (0.65–0.77)	0.86 (0.81–0.91)	0.90 (0.86–0.94)	0.77 (0.71–0.83)	0.89 (0.85–0.93)	0.88 (0.83–0.93)	0.91 (0.87–0.95)	0.84 (0.79–0.89)
	**AUC**		0.86 (0.81–0.91)	0.87 (0.82–0.92)	0.95 (0.92–0.98)	0.83 (0.77–0.89)	0.93 (0.89–0.97)	0.95 (0.92–0.98)	0.86 (0.81–0.91)	0.95 (0.92–0.98)	0.94 (0.91–0.97)	0.96 (0.94–0.98)	0.92 (0.88–0.96)

Furthermore, the WOA-based feature selection was applied to the reduced dataset; the number of variables decreased to 9, yet the predictive performance significantly improved across most models. The results show in Table 5 indicate that CatBoost achieved the highest overall performance, with a testing accuracy, precision, recall, and F1-score all reaching 0.95, and MCC, Kappa, and AUC at 0.92, 0.91, and 0.96, respectively. Moreover, MLP, RF, and SVM demonstrated high and consistent performance in the reduced model, each achieving an accuracy of 0.94, while XGBoost followed closely with an accuracy of 0.93.

Overall, the reduced dataset demonstrated superior predictive capability compared to the full dataset, despite utilizing fewer features. These findings provide strong evidence that applying the WOA for feature selection not only reduces data dimensionality but also enhances model performance. Notably, CatBoost achieved the best results using only 9 features, outperforming all other classifiers. This highlights its strong ability to effectively handle the optimized reduced feature set for predicting VI in patients with T2D.

Fig 11 illustrates the AUC values for the 11 machine learning models using the optimal features selected by WOA across both the full and reduced datasets. The figure demonstrates that the reduced high-risk dataset, which utilized only 9 predictors compared with 14 in the full dataset, achieved superior discriminative performance across most classifiers. In the reduced dataset, CatBoost achieved the highest AUC of 0.96 (95% CI: 0.93–0.99), followed by RF, MLP, and SVM, each with an AUC of 0.95 (95% CI: 0.92–0.98), and XGBoost with an AUC of 0.94 (95% CI: 0.91–0.97). These findings support the effectiveness of WOA-based feature selection in enhancing model discrimination and improving predictive performance for VI prediction among patients with T2D.

**Fig 11:**
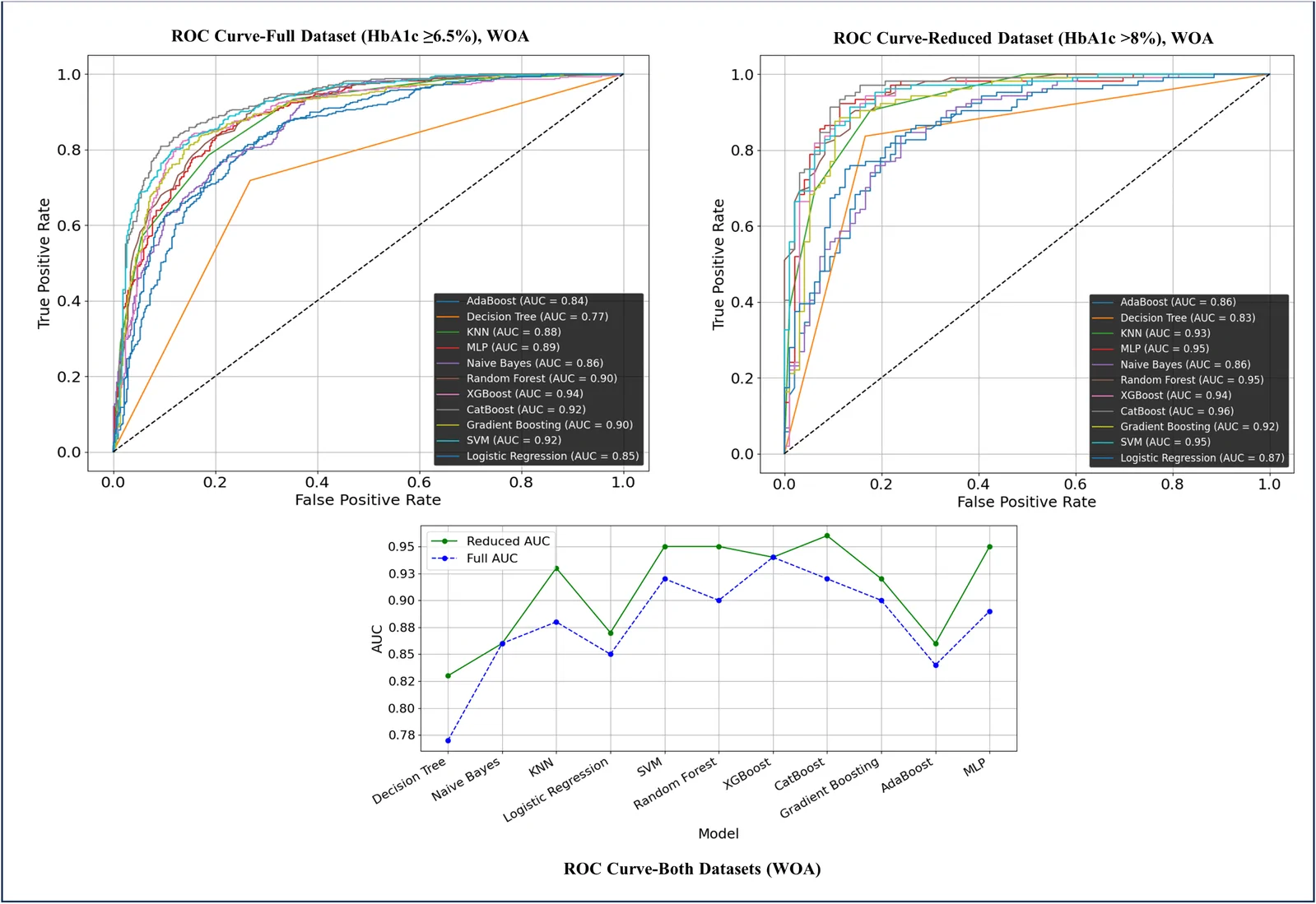
ROC curves of classification models with WOA-selected optimal features on full dataset (HbA1c ≥ 6.5%, number of variables = 14) and the reduced dataset (HbA1c > 8%, number of variables = 9).

### 5.6 Performance assessment of machine learning models with Black Widow Optimization (BWO) based feature selection in predicting visual impairment

The Black Widow Optimization (BWO) algorithm was applied to select the most significant predictors from the original 19 features, as shown in Figs 6 and 7. As shown in Table 6, 12 variables were selected for the full dataset (HbA1c ≥ 6.5%) and 10 variables for the reduced dataset (HbA1c > 8%), representing the most influential features contributing to VI prediction among patients with T2D. In the full dataset, RF achieved the highest testing accuracy of 0.90, with precision of 0.90, recall of 0.89, F1-score of 0.89, MCC of 0.77, Kappa of 0.78, and AUC of 0.89. The MLP also demonstrated competitive performance, with a testing accuracy of 0.88, precision of 0.87, recall of 0.87, F1-score of 0.87, MCC of 0.74, Kappa of 0.73, and AUC of 0.84. KNN followed closely with a testing accuracy of 0.87, precision of 0.87, recall of 0.86, F1-score of 0.87, MCC of 0.72, Kappa of 0.73, and AUC of 0.84. CatBoost and GraBoost each achieved a testing accuracy of 0.85, with AUC values of 0.86 for both. In the reduced dataset, RF again achieved the highest overall performance, with a testing accuracy of 0.92, precision of 0.91, recall of 0.91, F1-score of 0.91, MCC of 0.83, Kappa of 0.83, and AUC of 0.90. The MLP followed with a testing accuracy of 0.90, precision of 0.88, recall of 0.88, F1-score of 0.88, MCC of 0.77, Kappa of 0.79, and AUC of 0.85. Notably, KNN achieved a testing accuracy of 0.85 with the highest precision of 0.90 among all classifiers in the reduced dataset, alongside an AUC of 0.88, suggesting strong specificity in identifying true positive VI cases. CatBoost achieved an accuracy of 0.89 with an AUC of 0.86, and GraBoost an accuracy of 0.88 with an AUC of 0.88.

**Table 6:** Classification models with BWO-based feature selection for the full dataset (HbA1c ≥ 6.5%, number of variables = 12) and the reduced dataset (HbA1c > 8%, number of variables = 10).

			Models										
			Ada-Boost	LR	RF	DT	KNN	MLP	NB	SVM	XGBoost	CatBoost	GraBoost
**Training**	**Acc.**	**Full Model (HbA1c ≥ 6.5%)**	0.85 (0.82–0.88)	0.83 (0.80–0.86)	0.91 (0.88–0.93)	0.83 (0.80–0.86)	0.89 (0.87–0.92)	0.90 (0.88–0.93)	0.84 (0.81–0.87)	0.87 (0.84–0.90)	0.86 (0.83–0.89)	0.88 (0.85–0.91)	0.87 (0.84–0.90)
	**Prec.**		0.85 (0.82–0.88)	0.83 (0.80–0.86)	0.91 (0.88–0.93)	0.83 (0.80–0.86)	0.89 (0.87–0.92)	0.90 (0.88–0.93)	0.84 (0.81–0.87)	0.87 (0.84–0.90)	0.86 (0.83–0.89)	0.88 (0.85–0.91)	0.87 (0.84–0.90)
	**Recall**		0.84 (0.81–0.87)	0.82 (0.79–0.85)	0.90 (0.87–0.93)	0.82 (0.79–0.85)	0.88 (0.86–0.91)	0.89 (0.87–0.92)	0.83 (0.80–0.86)	0.86 (0.83–0.89)	0.85 (0.82–0.88)	0.87 (0.84–0.90)	0.86 (0.83–0.89)
	**F1-Score**		0.84 (0.81–0.87)	0.82 (0.79–0.85)	0.90 (0.87–0.93)	0.82 (0.79–0.85)	0.88 (0.86–0.91)	0.89 (0.87–0.92)	0.83 (0.80–0.86)	0.86 (0.83–0.89)	0.85 (0.82–0.88)	0.87 (0.84–0.90)	0.86 (0.83–0.89)
	**MCC**		0.70 (0.67–0.73)	0.67 (0.64–0.70)	0.80 (0.77–0.83)	0.67 (0.64–0.70)	0.77 (0.74–0.80)	0.79 (0.76–0.82)	0.69 (0.66–0.72)	0.75 (0.72–0.78)	0.74 (0.71–0.77)	0.76 (0.73–0.79)	0.75 (0.72–0.78)
	**κ**		0.69 (0.66–0.72)	0.66 (0.63–0.69)	0.79 (0.76–0.82)	0.66 (0.63–0.69)	0.76 (0.73–0.79)	0.78 (0.75–0.81)	0.68 (0.65–0.71)	0.74 (0.71–0.77)	0.73 (0.70–0.76)	0.75 (0.72–0.78)	0.74 (0.71–0.77)
	**AUC**		0.88 (0.85–0.91)	0.91 (0.88–0.94)	0.95 (0.92–0.97)	0.84 (0.81–0.87)	0.93 (0.90–0.96)	0.94 (0.91–0.97)	0.90 (0.87–0.93)	0.90 (0.87–0.93)	0.89 (0.86–0.92)	0.91 (0.88–0.94)	0.90 (0.87–0.93)
**Testing**	**Acc.**	**Full Model** **(HbA1c ≥ 6.5%)**	0.83 (0.78–0.88)	0.81 (0.76–0.86)	0.90 (0.85–0.94)	0.79 (0.74–0.84)	0.87 (0.82–0.92)	0.88 (0.83–0.93)	0.82 (0.77–0.87)	0.84 (0.79–0.89)	0.82 (0.77–0.87)	0.85 (0.80–0.90)	0.85 (0.80–0.90)
	**Prec.**		0.83 (0.78–0.88)	0.80 (0.75–0.85)	0.90 (0.85–0.94)	0.79 (0.74–0.84)	0.87 (0.82–0.92)	0.87 (0.82–0.92)	0.80 (0.75–0.85)	0.83 (0.78–0.88)	0.82 (0.77–0.87)	0.85 (0.80–0.90)	0.84 (0.79–0.89)
	**Recall**		0.82 (0.77–0.87)	0.80 (0.75–0.85)	0.89 (0.84–0.93)	0.79 (0.74–0.84)	0.86 (0.81–0.91)	0.87 (0.82–0.92)	0.75 (0.70–0.80)	0.78 (0.73–0.83)	0.81 (0.76–0.86)	0.84 (0.79–0.89)	0.84 (0.79–0.89)
	**F1-Score**		0.82 (0.77–0.87)	0.74 (0.69–0.79)	0.89 (0.84–0.93)	0.78 (0.73–0.83)	0.87 (0.82–0.92)	0.87 (0.82–0.92)	0.77 (0.72–0.82)	0.79 (0.74–0.84)	0.81 (0.76–0.86)	0.84 (0.79–0.89)	0.84 (0.79–0.89)
	**MCC**		0.67 (0.60–0.74)	0.58 (0.51–0.65)	0.77 (0.70–0.84)	0.60 (0.53–0.67)	0.72 (0.65–0.79)	0.74 (0.67–0.81)	0.59 (0.52–0.66)	0.63 (0.56–0.70)	0.63 (0.56–0.70)	0.67 (0.60–0.74)	0.68 (0.61–0.75)
	**κ**		0.66 (0.59–0.73)	0.57 (0.50–0.64)	0.78 (0.71–0.85)	0.59 (0.52–0.66)	0.73 (0.66–0.80)	0.73 (0.66–0.80)	0.62 (0.55–0.69)	0.62 (0.55–0.69)	0.62 (0.55–0.69)	0.66 (0.59–0.73)	0.67 (0.60–0.74)
	**AUC**		0.78 (0.73–0.83)	0.85 (0.80–0.90)	0.89 (0.86–0.95)	0.73 (0.68–0.78)	0.84 (0.79–0.89)	0.84 (0.79–0.89)	0.82 (0.77–0.87)	0.82 (0.77–0.87)	0.84 (0.79–0.89)	0.86 (0.81–0.91)	0.86 (0.81–0.91)
**Training**	**Acc.**	**Reduced Model (HbA1c > 8%)**	0.85 (0.82–0.88)	0.85 (0.82–0.88)	0.94 (0.89–0.95)	0.84 (0.81–0.87)	0.91 (0.88–0.94)	0.92 (0.89–0.95)	0.85 (0.82–0.88)	0.87 (0.84–0.90)	0.87 (0.84–0.90)	0.89 (0.86–0.92)	0.88 (0.85–0.91)
	**Prec.**		0.85 (0.82–0.88)	0.85 (0.82–0.88)	0.92 (0.89–0.95)	0.83 (0.80–0.86)	0.90 (0.87–0.93)	0.92 (0.89–0.95)	0.85 (0.82–0.88)	0.87 (0.84–0.90)	0.87 (0.84–0.90)	0.89 (0.86–0.92)	0.88 (0.85–0.91)
	**Recall**		0.85 (0.82–0.88)	0.85 (0.82–0.88)	0.91 (0.88–0.94)	0.81 (0.78–0.84)	0.90 (0.87–0.93)	0.91 (0.88–0.94)	0.86 (0.83–0.89)	0.87 (0.84–0.90)	0.89 (0.86–0.92)	0.90 (0.87–0.93)	0.89 (0.86–0.92)
	**F1-Score**		0.85 (0.82–0.88)	0.85 (0.82–0.88)	0.91 (0.88–0.94)	0.84 (0.81–0.87)	0.90 (0.87–0.93)	0.92 (0.89–0.95)	0.86 (0.83–0.89)	0.87 (0.84–0.90)	0.88 (0.85–0.91)	0.89 (0.86–0.92)	0.89 (0.86–0.92)
	**MCC**		0.71 (0.68–0.74)	0.70 (0.67–0.73)	0.83 (0.80–0.86)	0.66 (0.63–0.69)	0.80 (0.77–0.83)	0.83 (0.80–0.86)	0.72 (0.69–0.75)	0.76 (0.73–0.79)	0.78 (0.75–0.81)	0.79 (0.76–0.82)	0.78 (0.75–0.81)
	**κ**		0.70 (0.67–0.73)	0.69 (0.66–0.72)	0.82 (0.79–0.85)	0.65 (0.62–0.68)	0.79 (0.76–0.82)	0.82 (0.79–0.85)	0.71 (0.68–0.74)	0.75 (0.72–0.78)	0.77 (0.74–0.80)	0.78 (0.75–0.81)	0.77 (0.74–0.80)
	**AUC**		0.87 (0.84–0.90)	0.86 (0.83–0.89)	0.95 (0.90–0.96)	0.85 (0.82–0.88)	0.91 (0.88–0.94)	0.93 (0.90–0.96)	0.87 (0.84–0.90)	0.89 (0.86–0.92)	0.90 (0.87–0.93)	0.91 (0.88–0.94)	0.90 (0.87–0.93)
**Testing**	**Acc.**	**Reduced Model (HbA1c > 8%)**	0.85 (0.80–0.90)	0.84 (0.79–0.89)	**0.92** (0.87–0.96)	0.82 (0.77–0.87)	0.90 (0.85–0.95)	0.90 (0.85–0.95)	0.85 (0.80–0.90)	0.87 (0.82–0.92)	0.87 (0.82–0.92)	0.89 (0.84–0.94)	0.88 (0.83–0.93)
	**Prec.**		0.85 (0.80–0.91)	0.84 (0.79–0.89)	0.91 (0.86–0.95)	0.81 (0.76–0.86)	0.90 (0.85–0.95)	0.88 (0.83–0.93)	0.85 (0.80–0.90)	0.87 (0.82–0.92)	0.87 (0.82–0.92)	0.88 (0.83–0.93)	0.88 (0.83–0.93)
	**Recall**		0.85 (0.80–0.90)	0.84 (0.79–0.89)	0.91 (0.86–0.95)	0.81 (0.76–0.86)	0.88 (0.83–0.93)	0.89 (0.84–0.94)	0.81 (0.76–0.86)	0.87 (0.82–0.92)	0.87 (0.82–0.92)	0.89 (0.84–0.94)	0.88 (0.83–0.93)
	**F1-Score**		0.85 (0.80–0.90)	0.79 (0.74–0.84)	0.91 (0.86–0.95)	0.75 (0.70–0.80)	0.88 (0.83–0.93)	0.88 (0.83–0.93)	0.81 (0.76–0.86)	0.87 (0.82–0.92)	0.8 (0.82–0.92)	0.88 (0.83–0.93)	0.88 (0.83–0.93)
	**MCC**		0.71 (0.61–0.81)	0.67 (0.60–0.74)	0.83 (0.76–0.90)	0.61 (0.54–0.68)	0.77 (0.70–0.84)	0.78 (0.71–0.85)	0.64 (0.57–0.71)	0.73 (0.66–0.80)	0.75 (0.68–0.82)	0.76 (0.69–0.83)	0.75 (0.68–0.82)
	**κ**		0.69 (0.61–0.77)	0.65 (0.58–0.72)	0.83 (0.76–0.90)	0.63 (0.56–0.70)	0.79 (0.72–0.86)	0.79 (0.72–0.86)	0.62 (0.55–0.69)	0.73 (0.66–0.80)	0.74 (0.67–0.81)	0.86 (0.81–0.91)	0.84 (0.79–0.89)
	**AUC**		0.88 (0.87–0.90)	0.88 (0.87–0.90)	0.90 (0.87–0.93)	0.86 (0.85–0.88)	0.88 (0.84–0.94)	0.85 (0.80–0.92)	0.89 (0.85–0.93)	0.88 (0.83–0.93)	0.88 (0.83–0.93)	0.86 (0.83–0.91)	0.88 (0.85–0.94)

Overall, the reduced dataset yielded superior predictive performance compared with the full dataset despite utilizing fewer features, suggesting that BWO-based feature selection effectively identifies informative predictors for VI risk prediction among patients with T2D.

Fig 12 presents the ROC curves and AUC comparisons for all 11 classifiers across both datasets using BWO-selected features. RF achieved the highest AUC in both the full dataset (0.89, 95% CI: 0.86–0.95) and the reduced dataset (0.90, 95% CI: 0.87–0.93), with the reduced dataset demonstrating marginally superior discriminative performance across most classifiers, further supporting the effectiveness of BWO-based feature selection for VI risk prediction among patients with T2D.

**Fig 12:**
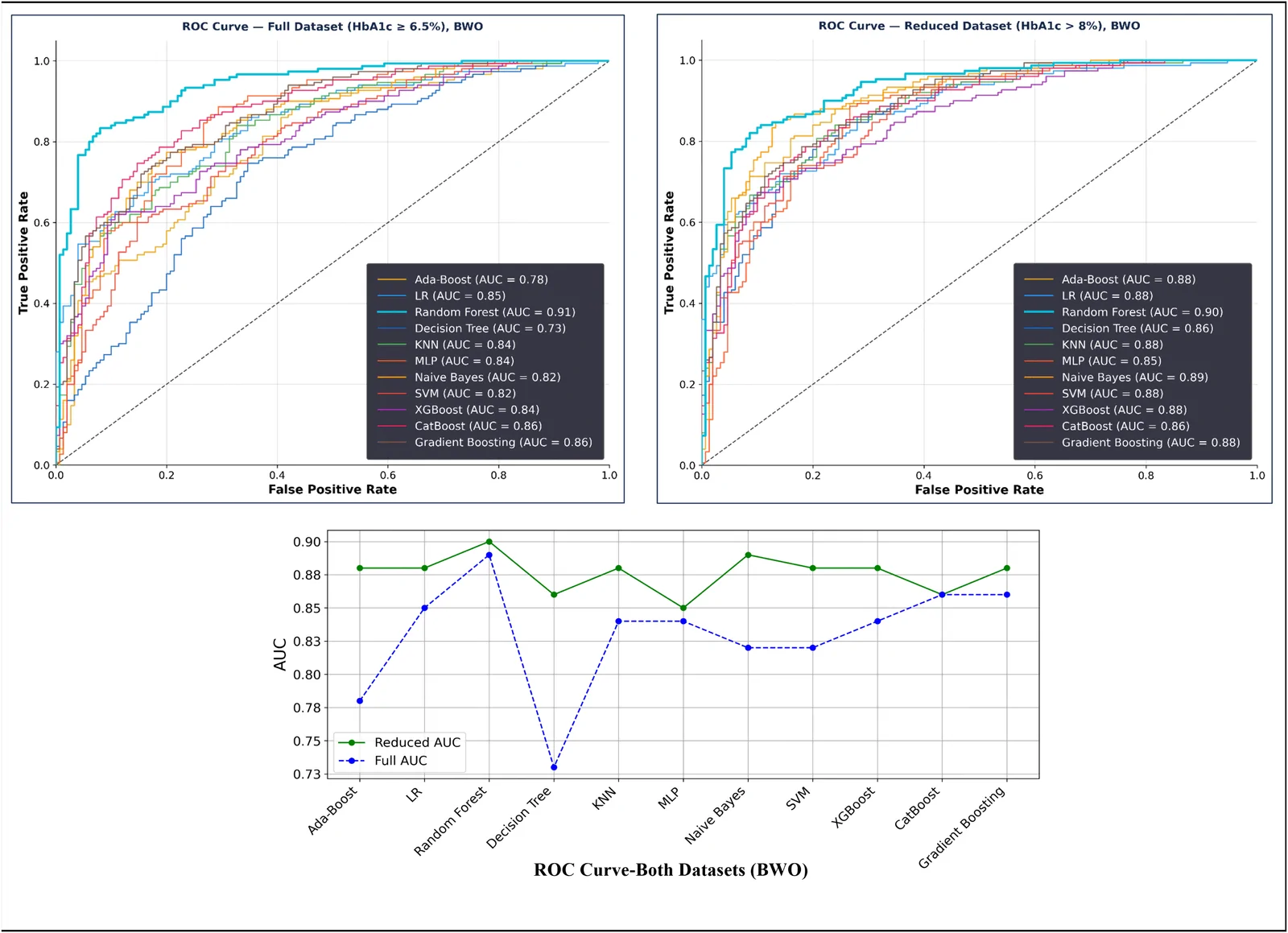
ROC curves of classification models with BWO-selected optimal features on full dataset (HbA1c ≥ 6.5%, number of variables = 12) and the reduced dataset (HbA1c > 8%, number of variables = 10).

## 6. Significant predictors of visual impairment in each model

Table 7 presents the optimal predictive variables identified by the best-performing model under each approach: baseline classification, WOA, and BWO, all evaluated on the reduced dataset (HbA1c > 8%). For the baseline classification approach, the MLP model identified 13 influential predictors of VI: gender, BMI, occupation, smoking, physical activity, type of food, HbA1c, hypertension, WBC, HDL, TG, vitamin D, and age. Under the WOA approach, the CatBoost model identified 9 key predictors: gender, BMI, occupation, smoking, type of food, HbA1c, HDL, TG, and age. Under the BWO approach, the RF model identified 10 key predictors: BMI, occupation, smoking, type of food, HbA1c, hypertension, WBC, TG, vitamin D, and age. Fig 13 presents the SHAP summary plots for the three best-performing models: the baseline-MLP, WOA-CatBoost, and BWO-RF. In the baseline- MLP model, TG emerged as the most influential predictor, followed by HbA1c, age, smoking, occupation, type of food, BMI, and HDL. In contrast, vitamin D, gender, physical activity levels, hypertension, and WBC demonstrated comparatively lower contributions. In the WOA-CatBoost model, HbA1c ranked as the dominant predictor, followed by TG, age, type of food, occupation, smoking, and BMI. Gender and HDL demonstrated the lowest contributions among all features. In the BWO-RF model, HbA1c remained the leading predictor, followed by TG, type of food, age, occupation, BMI, and smoking. Conversely, WBC, hypertension, and vitamin D demonstrated the lowest contributions among all features.

**Table 7:** Significant predictive variables selected by the best model for the reduced dataset under each method (Baseline, WOA, and BWO).

	Optimal features selected																			No. of features
	Gender	BMI	Marital Status	Nationality	Occupation	Smoking	Physical Activity	Type of Food	HbA1c	Hypertension	WBC	HDL	TC	TG	Vitamin D	Ferritin	SBP	DBP	Age	
Baseline -MLP	√	√			√	√	√	√	√	√	√	√		√	√				√	13
WOA-CatBoost	√	√			√	√		√	√			√		√					√	9
BWO- RF		√			√	√		√	√	√	√			√	√				√	10

**Fig 13:**
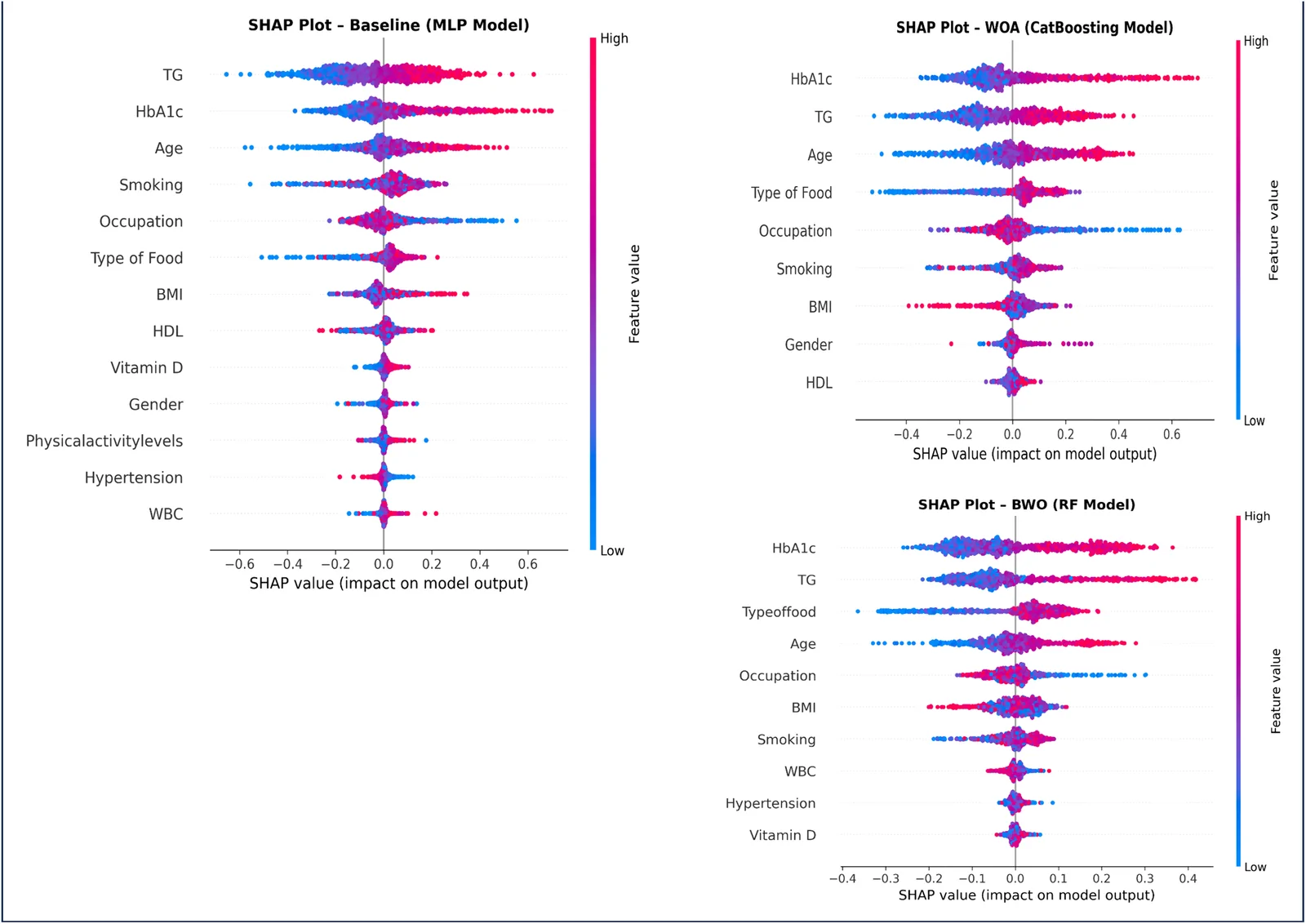
Key predictors identified across baseline, WOA, and BWO models. The SHAP summary plots display the mean absolute SHAP values on the y-axis, ranking features by their importance in each model. Each dot represents an individual’s SHAP value for a given feature, and vertical stacking of dots indicates areas of higher sample density. The color gradient reflects the original feature value, with red indicating high values and blue indicating low values.

Overall, HbA1c, TG, age, type of food, occupation, smoking, and BMI emerged as the most consistently influential predictors across all three best-performing models, where HbA1c and TG appeared as the top-ranked features in the WOA-CatBoost and BWO-RF models, while TG led in the baseline-MLP. Pairwise Spearman correlation analysis among these seven core features (S1 Fig) showed only weak correlations (|ρ| ≤ 0.31), indicating limited collinearity among the selected variables.These findings suggest that this core set of variables represents clinically meaningful predictors for VI risk among patients with T2D.

## 7. Performance comparison across baseline, WOA, and BWO Models

Table 8 presents a summary of the evaluation results for the best-performing model under each method, including baseline classification, WOA, and BWO, based on the identified feature set in the reduced dataset (HbA1c > 8%). In the baseline classification method, the optimal model was MLP, which utilized 13 features. This model achieved an accuracy of 0.91, precision of 0.92, recall of 0.93, F1-score of 0.92, MCC of 0.88, kappa of 0.86, and AUC of 0.94. For the WOA method, the outpreforming model was CatBoost with an accuracy of 0.95, precision of 0.95, recall of 0.95, F1-score of 0.95, MCC of 0.92, kappa of 0.91, and AUC of 0.96 using 9 features, and it recorded. In the BWO method, the best-performing model was RF, based on a reduced subset of 10 features, achieving an accuracy of 0.92, precision of 0.91, recall of 0.91, F1-score of 0.91, MCC of 0.83, kappa of 0.83, and AUC of 0.90. Overall, these performance metrics reflect each model’s ability to accurately predict the risk of VI in patients with T2D using the significant feature derived from the reduced dataset (HbA1c > 8%), as illustrated in Fig 14.

**Table 8:** Comparison of evaluation metrics for the best models under baseline, WOA, and BWO, including the number of significant variables.

Models	No. of features	Acc.	Prec.	Recall	F1-sore	MCC	κ	AUC
Baseline-MLP	13	0.91	0.92	0.93	0.92	0.88	0.86	0.94
WOA-CatBoost	9	0.95	0.95	0.95	0.95	0.92	0.91	0.96
BWO-RF	10	0.92	0.91	0.91	0.91	0.83	0.83	0.90

**Fig 14:**
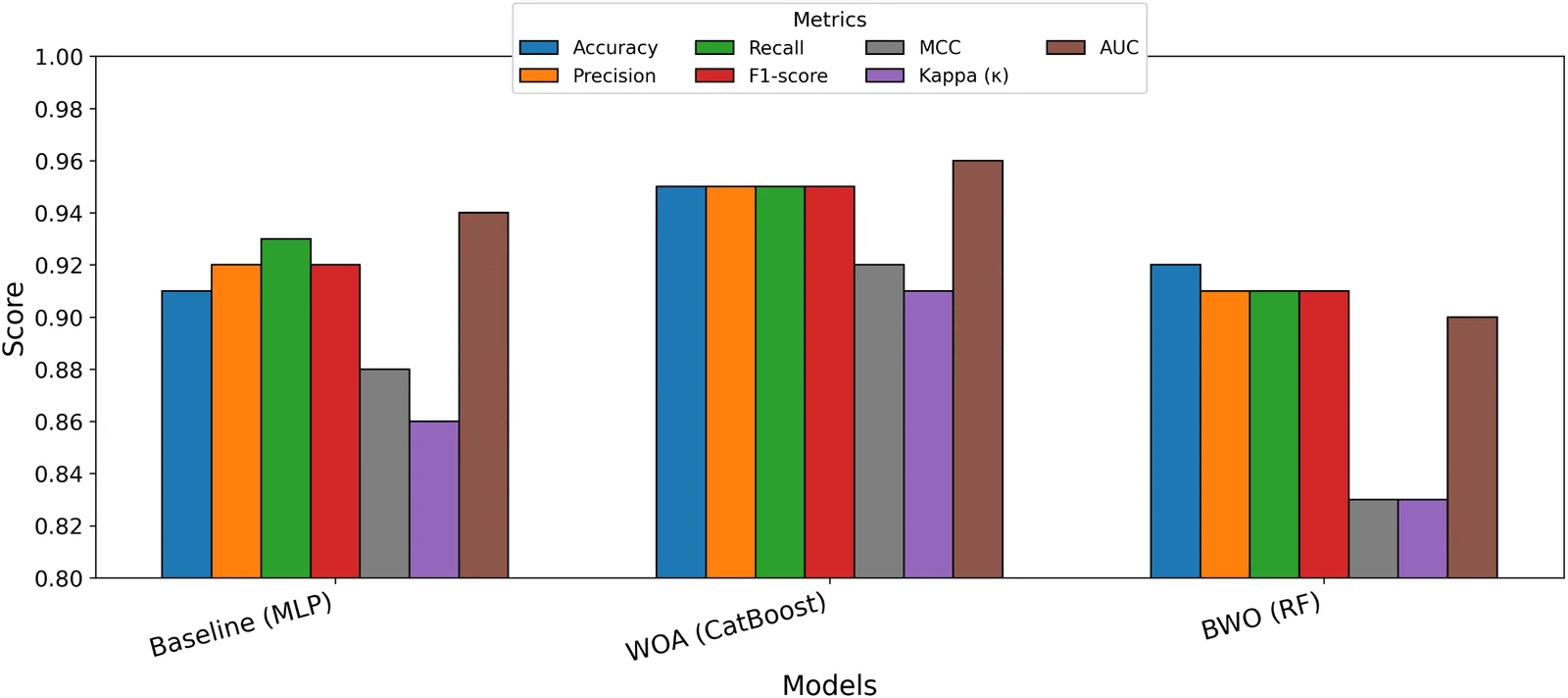
Bar chart comparing evaluation metrics for the best models selected under baseline, WOA, and BWO based on the identified feature for the reduced dataset (HbA1c > 8%).

To assess and statistically compare the performance of all models, a paired *t*-test was conducted across the five cross-validation folds to determine whether differences in model performance were statistically significant. Let $d_{i}=\;M_{A,i}-M_{B,i}$, where $M_{A,i}$ and $M_{B,i}$ denote the performance measures of Models A and B in fold i, respectively. The test evaluates whether the mean difference $d_{i}$ is statistically different from zero:

$H_{\text{0}}{:\mu }_{d}=\text{0}$, indicating no average difference in performance between the two models.

$H_{\text{1}}{:\mu }_{d}\text{≠}\text{0}$, indicating an average difference in performance between the two models.

To control for Type I error inflation across multiple pairwise comparisons, the Bonferroni correction was applied, yielding an adjusted significance threshold of α = 0.017 (0.05 ÷ 3). As shown in Table 9, only one of the three pairwise comparisons was statistically significant. Specifically, WOA-CatBoost significantly outperformed BWO-RF (t = 10.17, p < 0.001), demonstrating a clear and consistent accuracy advantage across all five cross-validation folds. In contrast, the comparisons between WOA-CatBoost and baseline-MLP (t = 2.53, p = 0.064), and between baseline-MLP and BWO-RF (t = 0.48, p = 0.655), did not reach the Bonferroni-adjusted significance threshold. The large t-value observed for the WOA-CatBoost versus BWO-RF comparison reflects the highly consistent fold-wise performance advantage of WOA, resulting in low within-pair variance and a correspondingly high-test statistic. This finding supports the robustness and stability of the WOA-CatBoost model as the classifier that performed best descriptively in this study.

**Table 9:** Pairwise statistical comparison of model accuracy using paired t-tests.

Comparison	t-stat	P-value	Result
WOA-CatBoost vs Baseline-MLP	2.53	0.064	Non-significant
WOA-CatBoost vs BWO-RF	10.17	< 0.001	Significant
Baseline-MLP vs BWO-RF	0.48	0.655	Non-significant

To strengthen the statistical evaluation, the DeLong non-parametric test was applied to compare AUC values between the three best-performing models on the held-out test set (20%). WOA-CatBoost achieved the highest AUC (0.96, 95% CI: 0.94–0.98), followed by baseline-MLP (0.94, 95% CI: 0.91–0.97) and BWO-RF (0.90, 95% CI: 0.87–0.93). Applying the Bonferroni correction (α = 0.017), the pairwise DeLong test results, presented in Table 10, showed that only the comparison between WOA-CatBoost and BWO-RF reached statistical significance (ΔAUC = +0.06, z = +4.000, p < 0.001), indicating a clear discriminative advantage of WOA-CatBoost over BWO-RF. The comparison between baseline-MLP and WOA-CatBoost (ΔAUC = −0.02, z = −1.176, p = 0.239) and between baseline-MLP and BWO-RF (ΔAUC = +0.04, z = +2.353, p = 0.019) did not reach Bonferroni-corrected significance, as both p-values exceeded the adjusted threshold of 0.017.

**Table 10:** Pairwise DeLong test comparison of AUC among the three best-performing models.

Comparison	Δ AUC	SE	z-stat	p-value	Result
Baseline-MLP vs WOA-CatBoost	−0.02	0.017	−1.176	0.239	Non-significant
Baseline-MLP vs BWO-RF	+0.04	0.017	+2.353	0.019	Non-significant
WOA-CatBoost vs BWO-RF	+0.06	0.015	+4.000	< 0.001	Significant

Calibration analysis confirmed that all three models were well calibrated, suggesting that the reported performance metrics are unlikely to be driven by substantial overfitting. WOA-CatBoost showed the strongest calibration performance (Brier Score = 0.060, ECE = 0.010, train–test accuracy gap = 0.01, AUC gap = 0.02), with the smallest calibration errors and minimal train–test discrepancy, indicating close agreement between predicted probabilities and observed outcomes. Baseline-MLP demonstrated good calibration performance (Brier Score = 0.100, ECE = 0.022, train–test accuracy gap = 0.04, AUC gap = 0.03), with modest calibration error and limited evidence of overfitting. BWO-RF showed comparatively weaker but still acceptable calibration (Brier Score = 0.120, ECE = 0.042, train–test accuracy gap = 0.02, AUC gap = 0.05), with a larger AUC gap reflecting greater train–test discrepancy relative to the other two models, as shown in S3 Table.

To further assess the potential influence of class imbalance on the reported classification performance, the no-information rate (NIR), defined as the accuracy achievable by always predicting the majority class, was calculated separately for each dataset. In the full dataset (HbA1c ≥ 6.5%), the majority class comprised patients with VI (55.5%; 555/1000), yielding an NIR of 0.555. In the reduced dataset (HbA1c > 8%), the higher baseline prevalence of VI (68.15%) yielded an NIR of 0.6815. A one-sample proportion (z) test was used to formally test whether model accuracy exceeded these baselines. In the full dataset, the best-performing model (WOA-XGBoost, accuracy = 0.92, n_test = 200) significantly exceeded the NIR (z = 10.39, p < 0.001). In the reduced dataset, WOA-CatBoost (accuracy = 0.95, n_test = 108) similarly exceeded its higher NIR (z = 5.99, p < 0.001). This is further reinforced by the Kappa statistic reported in Table 8, which is intrinsically adjusted for the agreement expected by chance under the observed class distribution; the substantial-to-almost-perfect κ values obtained (κ = 0.86–0.92 across models) corroborate that classification performance in both cohorts reflects genuine predictive signal rather than an artefact of class imbalance.

Collectively, multiple complementary statistical findings support WOA-CatBoost as the overall strongest-performing model in this study. First, the paired t-test on 5-fold cross-validation accuracy demonstrated a statistically significant and consistent accuracy advantage of WOA-CatBoost over BWO-RF (t = 10.17, p < 0.001, Bonferroni-corrected; Table 9). Second, the pairwise DeLong AUC test confirmed a statistically significant discriminative advantage of WOA-CatBoost over BWO-RF (ΔAUC = +0.06, z = +4.000, p < 0.001, Bonferroni-corrected; Table 10). Third, calibration metrics further supported the robustness of WOA-CatBoost, which achieved the lowest Brier Score, the lowest Expected Calibration Error (ECE), and the smallest train–test performance gaps, indicating superior probability calibration and minimal overfitting. Fourth, WOA-CatBoost consistently demonstrated the strongest overall predictive profile across all six performance metrics (Accuracy = 0.95, Precision = 0.95, Recall = 0.95, F1 = 0.95, MCC = 0.92, κ = 0.91; Table 8). These results were achieved using only 9 of the 19 original features, highlighting the effectiveness of WOA-based feature selection in reducing model complexity while improving predictive performance for VI risk prediction in patients with T2D. While the best-performing WOA model retained 9 features, HbA1c, TG, age, type of food, occupation, smoking, and BMI were consistently identified as influential predictors across all three best-performing models, suggesting that these variables constitute a robust and clinically relevant core feature set for VI risk prediction.

## 8. Discussion

To the best of our knowledge, this is the first study conducted in Saudi Arabia to utilize a local dataset to predict visual impairment (VI) among patients with type 2 diabetes (T2D) using a comprehensive evaluation of 11 machine learning classification algorithms, including Adaptive Boosting (AdaBoost), Logistic Regression (LR), Support Vector Machine (SVM), Decision Tree (DT), K-Nearest Neighbors (KNN), Random Forest (RF), Multi-Layer Perceptron (MLP), Naïve Bayes (NB), Extreme Gradient Boosting (XGBoost), Categorical Boosting (CatBoost), and Gradient Boosting (GraBoost). These models are applied both with and without nature-inspired feature Whale Optimization Algorithm (WOA) and Black Widow Optimization (BWO), to enhance model performance by selecting the most significant features. Model evaluation was carried out on two patient groups: a full cohort of individuals with HbA1c levels greater than or equal to 6.5% (full dataset) and a high-risk subgroup with HbA1c levels exceeding 8% (reduced dataset). To assess their effectiveness, model performance was evaluated using several metrics, including accuracy, precision, recall, F1-Score, Matthews Correlation Coefficient (MCC), Cohen’s Kappa (κ), area under the curve (AUC). This dual-cohort approach enhances both clinical relevance and interpretability. To ensure broad representation of the local diabetic population, the study used anonymized clinical data from 1,000 adults with T2D who received care at King Abdulaziz University Hospital (KAUH) in Jeddah, Saudi Arabia.

The findings of this study, as presented in Table 4, showed that among the baseline classification models without feature selection, the full dataset (HbA1c ≥ 6.5%) indicated that the MLP achieved the highest performance across all classifiers, with an accuracy = 0.88, precision = 0.89, recall = 0.89, F1-score = 0.89, MCC = 0.78, kappa = 0.76, and AUC = 0.90. In the reduced dataset (HbA1c > 8%), MLP also demonstrated the best performance across all classifiers, with an accuracy = 0.91, precision = 0.92, recall = 0.93, F1-score = 0.92, MCC = 0.88, kappa = 0.86, and AUC = 0.94. The most significant features identified in this model included HbA1c, TG, age, smoking status, type of food, BMI, HDL, gender, hypertension, occupation, vitamin D, physical activity, and WBC.

As shown in Fig 5, the WOA was applied to full dataset for feature selection. The features with the highest predictive power were 14 out of 19 input variables retained, including BMI, HbA1c, TG, type of food, age, SBP, HDL, vitamin-D, physical activity, marital status, occupation, gender, smoking, and TC. In the reduced dataset (HbA1c > 8%), the features with the highest predictive power in this subgroup were limited to 9 variables, namely HbA1c, HDL, type of food, age, BMI, TG, smoking, occupation and gender. Moreover, as presented in Table 5, the XGBoost model outperformed all other classifiers in the full dataset (HbA1c ≥ 6.5%), achieving an accuracy = 0.92, precision = 0.92, recall = 0.93, F1-score = 0.92, MCC = 0.88, kappa = 0.87, and AUC = 0.94. In the reduced dataset (HbA1c > 8%), CatBoost demonstrated the best performance, with identical metrics: accuracy, precision, recall, and F1-score all = 0.95, MCC = 0.92, kappa = 0.91, and AUC = 0.96 demonstrating the ability of WOA to preserve model accuracy with a significantly reduced feature set.

As illustrated in Figs 7 and 8, The BWO algorithm was employed to perform feature selection. In the full dataset (HbA1c ≥ 6.5%), 12 out of the original 19 variables were retained. These included HbA1c, HDL, physical activity, TG, BMI, smoking, type of food,SBP, TC, age, gender and occupation. In contrast, the reduced dataset (HbA1c > 8%) utilized only 10 predictors, namely HbA1c, TG, occupation, age, smoking, type of food, BMI, hypertension, WBC, vitamin-D, thereby achieving a more compact model while maintaining predictive strength. In terms of classification performance, as presented in Table 6, the RF model achieved the highest performance in the full dataset (HbA1c ≥ 6.5%), recording an accuracy = 0.90, precision = 0.90, recall = 0.89, F1-score = 0.89, MCC = 0.77, kappa = 0.78, and AUC = 0.89. In the reduced dataset (HbA1c > 8%), the RF outperformed all other classifiers, with accuracy = 0.92, precision = 0.91, recall = 0.91, F1-score = 0.91, MCC = 0.83, kappa = 0.83, and an AUC = 0.90.

As presented in Table 8, the study conducted a comparative analysis of evaluation metrics for the best-performing models under each method baseline classification, WOA, and BWO, including the number of significant predictive variables selected in each model. The MLP model under the classification approach demonstrated solid performance, relying on 13 significant predictive variables and achieving an accuracy of 0.91, precision of 0.92, recall of 0.93, F1-score of 0.92, MCC of 0.88, kappa of 0.86, and AUC of 0.94. In contrast, the CatBoost model optimized via the WOA algorithm retained only nine key variables while achieving the highest predictive accuracy of 0.95, with precision of 0.95, recall of 0.95, F1-score of 0.95, MCC of 0.92, kappa of 0.91, and AUC of 0.96. Meanwhile, the BWO-optimized RF model achieved an accuracy of 0.92, precision of 0.91, recall of 0.91, F1-score of 0.91, MCC of 0.83, kappa of 0.83, and AUC of 0.90 using 10 predictive variables, confirming the efficiency of nature-inspired optimization algorithms in constructing compact yet highly accurate models. To statistically validate the observed performance differences, a Bonferroni-corrected paired t-test was conducted across the five cross-validation folds at an adjusted significance threshold of α = 0.017. The results, summarized in Table 9, indicate that only the comparison between WOA-CatBoost and BWO-RF reached statistical significance (*t* = 10.17, *p* < 0.001), confirming a significant performance advantage of WOA-CatBoost over BWO-RF. In contrast, the comparisons between WOA-CatBoost and Baseline-MLP (*t* = 2.53, *p* = 0.064) and be*t*ween Baseline-MLP and BWO-RF (*t* = 0.48, *p* = 0.655) did no*t* reach the Bonferroni-adjusted threshold, suggesting no statistically significant difference between these pairs. To further validate discriminative performance, the DeLong test was applied on the held-out test set (Table 10). WOA-CatBoost achieved the highest AUC (0.96, 95% CI: 0.94–0.98), and its advantage over BWO-RF was statistically significant after Bonferroni correction (ΔAUC = +0.06, z = +4.000, p < 0.001). Overall, WOA-CatBoost achieved the highest predictive performance across all metrics, followed by BWO-RF, with WOA-CatBoost demonstrating a statistically confirmed superiority over BWO-RF in both accuracy and AUC. These findings demonstrate the effectiveness of nature-inspired optimization algorithms in reducing model complexity while maintaining strong predictive performance. In particular, WOA achieved the highest predictive accuracy using a smaller set of variables, resulting in a more efficient framework for visual impairment risk prediction among patients with T2D. It is important to interpret the higher predictive performance observed in the reduced dataset within the context of the clinically defined high-risk subgroup (HbA1c > 8%). As shown by the NIR analysis above, this subgroup has a higher baseline prevalence of visual impairment (NIR = 68.15% vs. 55.5% in the full dataset). Therefore, the improved performance should be interpreted within the context of this clinically relevant subgroup rather than being directly generalized to the broader T2D population. The proposed model is intended to support visual impairment risk stratification among patients with poor glycaemic control.

While the best-performing WOA-CatBoost model selected nine predictive variables, seven of these (HbA1c, TG, age, type of food, occupation, smoking, and BMI) were retained across all three best-performing models, despite differences in their underlying classifiers and feature-selection strategies. This convergence suggests that these variables represent a stable and clinically relevant predictor set for visual impairment risk in T2D patients. SHAP analysis further revealed that HbA1c and TG were the two most dominant predictors, with age, BMI, type of food, occupation, and smoking demonstrating meaningful contributions across all three models (Fig 13).

This highlights the robustness of this variable set as a clinically relevant core feature set for developing simplified yet powerful predictive models, thereby enhancing their applicability in routine clinical settings for VI risk prediction among patients with T2D in the Saudi population. The predictors selected by the best-performing models are clinically plausible and biologically consistent with the pathophysiology of diabetes-related visual complications. HbA1c is a marker of chronic hyperglycaemia and is strongly associated with retinal microvascular injury, endothelial dysfunction, and progression of diabetes-related ocular disease. Age may reflect cumulative exposure to metabolic and vascular damage, as well as a longer duration of diabetes and comorbidities. BMI is associated with insulin resistance, systemic inflammation, hypertension, and dyslipidaemia, which may accelerate microvascular complications. Elevated TG may contribute to lipid exudation, endothelial dysfunction, and retinal vascular damage. Smoking can increase oxidative stress and vascular inflammation, while physical inactivity and unhealthy dietary patterns may worsen glycaemic control and metabolic risk. Therefore, the selected feature set is not only statistically informative but also clinically interpretable.

From a biological perspective, the dominant importance of HbA1c and TG suggests a synergistic metabolic pathway underlying VI risk. Chronic hyperglycaemia, reflected by elevated HbA1c, is strongly associated with diabetic retinal injury through advanced glycation end-product accumulation, pericyte apoptosis, and disruption of the blood retinal barrier [63,64]. In the present study, HbA1c emerged as the dominant predictor in both optimized models and remained among the strongest predictors in the baseline model, reinforcing its central role in VI risk stratification. Moreover, the decision to stratify the cohort using HbA1c > 8% was clinically justified, as 68.15% of patients in the poor-control subgroup presented with VI, compared with only 2.23% in the well-controlled group (HbA1c < 7%), further supporting the clinical importance of persistent glycaemic dysregulation [16]. In addition, TG emerged as a highly recurrent lipid predictor across all three models, consistent with evidence that dyslipidaemia contributes to retinal vascular injury through lipoprotein deposition, oxidative stress, and impaired vascular autoregulation [65]. In the present cohort, VI prevalence rose progressively with TG severity, from 41.92% among patients with normal TG to 56.31% among those with moderately high TG and 59.73% among those with high TG, providing descriptive support for its repeated selection. Collectively, the recurrent co-selection of HbA1c and TG suggests that glycaemic and lipid dysregulation jointly represent the principal metabolic predictors of VI risk in this population. BMI also demonstrated consistent importance across models, reflecting its established association with insulin resistance, chronic inflammation, and metabolic dysregulation [66,67]. Supporting this interpretation, VI prevalence increased markedly across BMI categories, from 21.74% among normal-weight patients to 58.46% among overweight and 67.39% among obese patients, reinforcing its predictive relevance. Age also remained a recurrent predictor [68]. Although VI prevalence was broadly similar across age groups in the descriptive analysis (54.3–59.5%), its repeated selection by the machine learning models suggests that age may contribute through complex interactions with other metabolic risk factors rather than through an independent linear increase in VI prevalence. Occupation also emerged as a consistent predictor. Although not a direct biological factor, occupation may act as a proxy for broader socioeconomic determinants, including health literacy, healthcare access, healthcare utilization, and lifestyle behaviours [27]. Given that 71.2% of the overall cohort were unemployed, this variable may reflect structural vulnerability that influences long-term disease management beyond conventional biomarkers. Type of food also demonstrated consistent predictive importance across the optimized models. Notably, VI prevalence was higher among patients reporting healthy dietary patterns (64.90%) than among those with unhealthy patterns (50.38%). This unexpected finding may reflect reverse causation, whereby patients with established diabetes complications, including VI, adopted healthier dietary habits following medical advice after diagnosis. Consequently, the predictive contribution of dietary patterns may primarily arise through interactions with other metabolic variables such as HbA1c, TG, and BMI rather than as an isolated independent effect [69,70]. Similarly, VI prevalence was higher among non-smokers (64.89%) than smokers (54.09%) in the descriptive analysis. This unexpected pattern may reflect smoking cessation following disease diagnosis, survivor bias, or residual confounding, rather than indicating a protective effect of smoking. Accordingly, its contribution to the predictive models is more likely to reflect interaction-dependent predictive effects than a direct independent association, consistent with the mixed findings reported in previous studies [34,37].

Our findings are broadly consistent with previous ML and epidemiological studies reporting HbA1c, age, BMI, diabetes duration, and lipid-related variables as important predictors of diabetic retinopathy or diabetes-related visual complications [17,18]. However, several differences should be noted. Some previous studies reported Random Forest or XGBoost as the best-performing models [13,38], whereas WOA-CatBoost achieved the strongest performance in the present study. This difference may be explained by the ability of CatBoost to handle mixed clinical and categorical predictors, combined with WOA-based feature selection that reduced irrelevant or redundant variables. In addition, many previous studies focused specifically on diabetic retinopathy, while the present study focused on visual impairment among patients with T2D. Differences in outcome definition, population characteristics, healthcare setting, predictor availability, and validation strategy may therefore explain variation in selected predictors and model performance across studies. In contrast, other studies [10,12] emphasised sociodemographic and lifestyle determinants such as education level, income, and cardiovascular history as primary risk factors, further reflecting the heterogeneity of predictor importance across different population contexts.

Some findings differed from previous studies. For example, diabetes duration, insulin therapy, and hypertension have been reported as dominant predictors of diabetic retinopathy in several cohorts, whereas these factors were not consistently retained among the strongest predictors in our final selected models. This discrepancy may reflect differences in the clinical outcome studied, because visual impairment is broader than diabetic retinopathy and may arise from multiple ocular and systemic pathways. It may also reflect differences in sample composition, missingness patterns, local treatment practices, and feature-selection procedures. Therefore, the absence of some conventional risk factors from the final reduced feature set should not be interpreted as evidence that they are clinically unimportant, but rather that they added limited incremental predictive value in the present dataset. Importantly, the recurrent selection of these metabolic and lifestyle-related variables by two independent optimization algorithms (WOA and BWO), despite their differing search mechanisms, strengthens confidence that these predictors reflect genuine pathophysiological signals rather than statistical artefacts.

Regarding model performance, the WOA-CatBoost model achieved an accuracy of 0.95 and an AUC of 0.96, demonstrating highly competitive predictive performance relative to previously published machine learning studies on diabetic ocular complications. For example, a random forest-based diabetic retinopathy (DR) prediction model reported an accuracy of 86% [38], while an XGBoost-based model achieved an AUROC of 0.82 [13]. Similarly, a WOA-based ensemble classifier for diabetes prediction reported an accuracy of 83% [32]. Importantly, unlike prior studies that relied on specialist clinical settings [38], the proposed WOA-CatBoost model achieved this performance using only 9 routinely collected clinical variables, substantially lowering the barrier to implementation in primary care and resource-limited healthcare settings. This highlights the practical translational value of the proposed framework, particularly in environments where specialist ophthalmic screening infrastructure may be limited. The statistical advantage of WOA-CatBoost over BWO-RF was supported by both the Bonferroni-corrected paired t-test (t = 10.17, p < 0.001) and the DeLong AUC comparison (ΔAUC = +0.06, z = +4.000, p < 0.001). In addition, calibration analysis demonstrated strong model reliability (Brier Score = 0.060, ECE = 0.010, train–test accuracy gap = 0.01), suggesting that the observed performance reflects good generalisability with limited evidence of substantial overfitting. Collectively, these findings support the robustness of both the selected predictors and the WOA-CatBoost framework across different model configurations.

These findings enhance understanding of VI risk among patients with T2D in Saudi Arabia and demonstrate the value of integrating nature-inspired optimization with machine learning for patient-specific risk prediction. Unlike prior Saudi studies relying on single biomarkers or conventional statistical approaches [14,16,18,36,37], the proposed framework incorporates multiple interacting clinical and lifestyle variables within an optimized predictive model [71]. Importantly, the identified feature set (HbA1c, TG, age, type of food, occupation, smoking, and BMI) comprises routinely collected primary care variables, supporting the feasibility of integrating this tool into standard diabetic annual reviews for earlier identification of patients at high risk of VI.

Although the present study was conducted using data from King Abdulaziz University Hospital, a tertiary referral hospital in Jeddah, which may introduce referral bias by over-representing patients with more complex disease, poorer glycaemic control, multiple comorbidities, and established diabetes-related complications, the demographic and clinical characteristics of the study cohort were broadly comparable with those reported in published Saudi T2D populations. Comparison with published Saudi cohorts showed that the present study included 55.4% men and 78.0% of patients with hypertension, broadly comparable with a multi-region Saudi study reporting 51.1% men and approximately 79% with hypertension [72]. However, 66.5% of the present cohort were aged 60 years or older, compared with 39.7% in that study, and 82.1% had HbA1c ≥ 7%, compared with 76.7% with uncontrolled diabetes [72]. Furthermore, obesity was present in 45.7% of the present cohort, while 79.4% of participants were either overweight or obese, compared with 59.3% reported in a recent Saudi registry of 53,741 patients from 19 governmental hospitals and centres [73]. These findings suggest that the cohort reflects many of the demographic and cardiometabolic characteristics of Saudi adults with T2D while over-representing older patients and those with poorer glycaemic control, consistent with referral bias. Prospective external validation in independent cohorts from other Saudi regions, such as King Saud University Medical City in Riyadh or King Fahad Specialist Hospital in Dammam, is therefore warranted to establish the external validity of the proposed model and confirm its robustness and calibration in independent Saudi cohorts. Accordingly, any recommendations regarding screening, clinical decision support, or implementation should be considered pending external validation in multi-centre Saudi cohorts.

## 9. Limitations

While rigorous internal validation procedures were implemented, including stratified train–test splitting, 5-fold cross-validation, and calibration analysis, to minimize overfitting and strengthen internal robustness. Nevertheless, future external validation using independent multicenter cohorts is essential to confirm generalizability and clinical reproducibility. Future studies should also assess whether integrating ophthalmologic imaging, genetic markers, or inflammatory biomarkers provides incremental predictive value beyond routinely collected clinical variables, thereby supporting the development of more robust and clinically deployable decision-support tools for VI risk prediction. Also, the retrospective single-centre design may limit the generalizability of the findings beyond the studied population. Although the proposed framework was intentionally developed using routinely collected clinical variables to maximize practical applicability, the absence of complementary ophthalmologic imaging data (e.g., fundus photographs) may have limited additional diagnostic information. We also acknowledge that external validation was not performed; therefore, the transportability of the proposed model to independent populations remains uncertain. Furthermore, although measured confounders were considered, residual confounding from unmeasured clinical, behavioural, or socio-demographic factors cannot be entirely excluded.

## 10. Conclusion

Type 2 diabetes (T2D) remains a pressing global health issue, particularly due to its long-term complications such as visual impairment (VI), which significantly impact quality of life. In Saudi Arabia, where T2D prevalence is among the highest globally, the integration of data-driven prediction models represents a promising strategy to enhance early detection and intervention through monitoring a smaller number of associated features. This study is the first of its kind in Saudi Arabia to develop predictive models for VI among T2D patients using a comprehensive comparison of 11 machine learning classifiers. It also introduces the use of two nature-inspired feature selection algorithms Whale Optimization Algorithm (WOA) and Black Widow Optimization (BWO) to optimize model performance while reducing feature dimensionality.

The analysis was conducted on anonymized data from 1,000 patients at King Abdulaziz University Hospital, divided into two cohorts: a full dataset (HbA1c ≥ 6.5%) and a high risk reduced dataset (HbA1c > 8%). The findings demonstrated that models developed using the reduced high-risk dataset consistently achieved superior predictive performance. Among all evaluated approaches, the WOA-CatBoost model achieved the highest overall performance (accuracy = 0.95, AUC = 0.96), followed by the baseline-MLP model (accuracy = 0.91, AUC = 0.94) and the BWO-RF model (accuracy = 0.92, AUC = 0.90). Statistical comparisons further confirmed the superiority of WOA-CatBoost, particularly over BWO-RF, while calibration analysis demonstrated strong internal model reliability with limited evidence of overfitting. Importantly, WOA reduced the feature space from 19 variables to only 9 highly informative predictors, improving both predictive accuracy and model interpretability.

Across the three best-performing models, HbA1c and TG consistently emerged as the two most dominant predictors, with age, BMI, type of food, occupation, and smoking also demonstrating meaningful predictive relevance. These findings reinforce the importance of integrating metabolic, lifestyle, and demographic indicators when assessing VI risk among patients with T2D.

The proposed predictive framework enables healthcare professionals to make accurate, data-driven clinical decisions using a concise set of highly significant variables. By leveraging machine learning techniques supported by nature-inspired optimization algorithms, the models in this study demonstrate that high accuracy can be maintained while significantly reducing the number of required indicators. This enhances the practical applicability of the system in healthcare settings, allowing physicians to focus on the most important predictive factors for early detection and targeted intervention to reduce the risk of VI among patients with T2D in Saudi Arabia.

## Supporting information

S1 Table Summary of machine learning models used in the previous study [51]. (PDF)

S2 Table Grid search results and optimal hyperparameter configurations for all classification models across baseline, WOA, and BWO. (PDF)

S3 Table Calibration analysis for the three best-performing models. (PDF)

S1 File Mathematical Formulation of the Black Widow Optimization (BWO) Algorithm. (PDF)

S2 File Mathematical Formulation of the Whale Optimization Algorithm (WOA). (PDF)

S1 Fig Pairwise Spearman correlation heatmap of the final selected features in the reduced dataset (HbA1c > 8%). (TIF)
