# Supplementary material for: Predicting visual impairment in type 2 diabetes using key performance indicators identified by the Black Widow and Whale Optimization Algorithms: A case study in Saudi Arabia
Source: PLoS One. 2026 Oct 21;21(10):e0361022. doi: 10.1371/journal.pone.0361022 (PMC13686523; doi:10.1371/journal.pone.0361022)
Supplement: Supplementary file 1 [file pone.0361022.s001.pdf]

Table S1. Summary of machine learning models used in the previous study [51].

Model	Description
**Logistic Regression (LR)**	LR is a statistical method that examines the relationship between predictor variables and a binary response [41]. It is particularly valuable in healthcare, as it allows researchers to estimate the likelihood of outcomes based on key predictors [42]. A significant advantage of LR is its potential to process categorical variables, making it suitable for binary classification tasks. In this model, the probability of success is calculated using a logistic transformation of the independent variables, as illustrated in the equation (1): $p_i = \frac{\exp(\alpha_0+\alpha_1 z_{i1}+\alpha_2 z_{i2}+\cdots+\alpha_p z_{ip})}{1+\exp(\alpha_0+\alpha_1 z_{i1}+\alpha_2 z_{i2}+\cdots+\alpha_p z_{ip})}$ (1) Here, π_i represents the probability that a sample belongs to a specific category of the binary response variable, often referred to as the 'success probability', clearly, $0 \leq p_i \leq 1$, and the coefficients $\alpha_0, \alpha_1, \alpha_2, \ldots, \alpha_p$ correspond to the explanatory variables $z_{i1}, z_{i2}, \ldots, z_{ip}$.
**Decision Tree (DT)**	DT is a model utilized for classification when the target variable is categorical. The tree divides data based on predictor variables into root, internal, and leaf nodes [43]. It grows by minimizing impurity at each node using criteria such as Information Gain Ratio, Gain, and Gini Index [44]. The tree starts with all observations at the root node, and successive splits determine the importance of the predictor variables. The Gini Index selects splitting variables at internal nodes, optimizing the tree structure.<br>Here, D represents the dataset or the set of observations at a given node in the decision tree. Specifically, it refers to the subset of data points that reach the node during the tree-building process. C_i represents the class to which an observation in D may belong and p_i represents the likelihood of an observation within D being part of the class C_i, and it is calculated as shown in equation (2): $p_i = \frac{\lvert C_i, D\rvert}{\lvert D\rvert}$. (2)
**Random Forest (RF)**	RF is a collective technique that uses random selection to generate multiple decision trees from various variables and data subsets [45]. These trees are merged through a majority voting mechanism to create a powerful classifier. Tree depth, the number of predictors per tree, the total number of trees, and the minimal number of observations needed at each leaf node are important factors [46]. To assess performance, the dataset is separated into distinct training and testing sections. The main advantage of this approach is its capacity to assess the significance of variables using metrics such as the Gini Index and model accuracy declines [47].
**Support Vector Machine (SVM)**	SVM is primarily used in tasks related to classification and regression, making it a type of supervised learning model [48]. It identifies an optimal separating hyperplane, or maximum margin classifier, which maximizes the distance between different classes and minimizes classification errors [48]. SVM utilizes the kernel trick to perform non-linear classification, allowing it to efficiently process complex, high-dimensional datasets [49]. This capability makes SVM particularly valuable in fields requiring precise predictive modeling and risk minimization, such as medical diagnostics.
**Naive Bayes classifier (NB)**	The NB algorithm is a probabilistic classifier based on Bayes' Theorem, assuming strong feature independence. This model is effective in applications such as recommendation systems, predictive analytics, and spam filtering due to its simplicity and predictive accuracy [50]. The classification process involves the following four steps:<br>(i) Model Construction: Deriving sample mean (μ) and variance (σ^2) for numeric data and calculating probabilities for categorical data.

	(ii) Probability Calculation: Using the formula for each numeric datum, as demonstrated in equation (3):<br>$P(x_i\|y) = \frac{1}{\sqrt{2\pi\sigma^2}} exp\left(-\frac{(x_i-\mu_y)^2}{2\sigma_y^2}\right),$ (3)<br>where μ_y and σ_y^2 are the mean and variance for class y, respectively.<br>(iii) Final Probability: Computing the product of individual probabilities, as equation (4):<br>$P(X\|y) = \prod_{i=1}^{n} P(x_i\|y).$ (4)<br>(iv) Classification: Determining the class by comparing total probabilities, as equation (5):<br>$P(Y\|X) = \frac{P(Y)\prod_{i=1}^{n} P(x_i\|Y)}{P(X)}.$ (5)<br>This streamlined approach requires minimal training data, making naive bayes highly efficient for classification tasks.
**K-Nearest Neighbor Algorithm (KNN)**	K-NN is a simple, non-parametric technique that classifies or predicts new data by evaluating the proximity of data points [55]. It selects the number K of closest neighbors and calculates the distance to all training points using Euclidean or Manhattan metrics [56]. The new data point is then classified by majority rule among its K nearest neighbors or predicted by averaging the outcomes in regression tasks [57]. This efficient method leverages spatial proximity, making it suitable for classification and regression without an underlying model.
**Adaptive Boosting (ADA-Boost)**	AdaBoost is a collective method to enhance prediction performance by combining several weak models into a stronger one [58]. It modifies the importance of misclassified samples in each cycle, allowing subsequent models to concentrate on rectifying prior errors [59]. The influence of each weak model is based on its error rate., and the overall model is progressively refined through this iterative approach, making AdaBoost effective for challenging classification problems [60].
**Gradient Boosting (GraBoost)**	GB is an ML approach that improves model accuracy by adding weak models sequentially [61]. Each subsequent model focuses on correcting the errors of the prior one by employing a differentiable loss function to guarantee ongoing refinement. This technique is widely used for enhancing classification and prediction results [62].
**Extreme Gradient Boosting (XGBoost)**	XGBoost is an advanced gradient-boosting algorithm incorporating regularization methods to minimize overfitting [63]. It is recognized for its ability to scale, efficiency, and capacity to process numerical and categorical data. XGBoost utilizes parallel tree boosting to enhance differentiable loss functions, making it effective for tasks such as classification and regression [64]. Its robustness to outliers and flexibility in model tuning make it a preferred choice for advanced predictive analytics.
