# Supplementary material for: Predicting visual impairment in type 2 diabetes using key performance indicators identified by the Black Widow and Whale Optimization Algorithms: A case study in Saudi Arabia
Source: PLoS One. 2026 Oct 21;21(10):e0361022. doi: 10.1371/journal.pone.0361022 (PMC13686523; doi:10.1371/journal.pone.0361022)
Supplement: Supplementary file 2 [file pone.0361022.s002.pdf]

Table S2. Grid Search Results and Optimal Hyperparameter Configurations for All Classification Models Across Baseline, WOA, and BWO.

Baseline Models: Grid Search — Full Dataset (HbA1c >= 6.5%, 19 features)
==

Model	Best CV Score	Optimal Hyperparameters
AdaBoost	0.8299	algorithm=SAMME, learning_rate=1.0, n_estimators=100
Logistic Regression	0.8359	C=1, max_iter=200, solver=lbfgs
Random Forest	0.8576	max_depth=8, min_samples_split=10, n_estimators=80
Decision Tree	0.7938	max_depth=5, min_samples_split=10
KNN	0.8463	n_neighbors=9, p=1, weights=distance
MLP	0.8926	activation=tanh, hidden_layer_sizes=(50,), max_iter=200
Naive Bayes	0.8278	var_smoothing=1e-11
SVM	0.8480	C=1, kernel=rbf
XGBoost	0.8912	learning_rate=0.1, max_depth=7, n_estimators=80, subsample=0.8
CatBoost	0.8635	depth=5, iterations=150, learning_rate=0.1
Gradient Boosting	0.8254	learning_rate=0.1, max_depth=6, n_estimators=80

Baseline Models: Grid Search — Reduced Dataset (HbA1c > 8%, 19 Features)
==

Model	Best CV Score	Optimal Hyperparameters
AdaBoost	0.8518	algorithm=SAMME, learning_rate=0.8, n_estimators=70
Logistic Regression	0.8494	C=0.1, max_iter=200, solver=lbfgs
Random Forest	0.8998	max_depth=6, min_samples_split=5, n_estimators=120
Decision Tree	0.8318	max_depth=7, min_samples_split=30
KNN	0.8516	n_neighbors=9, p=2, weights=distance
MLP	0.9492	activation=tanh, hidden_layer_sizes=(50,), max_iter=300
Naive Bayes	0.8778	var_smoothing=1e-11
SVM	0.8316	C=0.1, kernel=linear

XGBoost	0.9039	learning_rate=0.1, max_depth=3, n_estimators=100, subsample=0.8
CatBoost	0.9016	depth=3, iterations=150, learning_rate=0.1
Gradient Boosting	0.8990	learning_rate=0.05, max_depth=2, n_estimators=100

Whale Optimization Algorithm (WOA) Models: Grid Search — Full Dataset (HbA1c >= 6.5%, 14 features)
==

Model	Best CV Score	Optimal Hyperparameters
AdaBoost	0.8431	algorithm=SAMME, learning_rate=0.8, n_estimators=70
Logistic Regression	0.8625	C=10, max_iter=200, solver=lbfgs
Random Forest	0.9087	max_depth=6, min_samples_split=5, n_estimators=100
Decision Tree	0.8388	max_depth=7, min_samples_split=10
KNN	0.8989	n_neighbors=9, p=1, weights=uniform
MLP	0.9018	activation=tanh, hidden_layer_sizes=(100,), max_iter=300
Naive Bayes	0.8698	var_smoothing=1e-11
SVM	0.9058	C=10, kernel=linear
XGBoost	0.9489	learning_rate=0.1, max_depth=7, n_estimators=100, subsample=0.9
CatBoost	0.9255	depth=5, iterations=200, learning_rate=0.04
Gradient Boosting	0.9096	learning_rate=0.05, max_depth=4, n_estimators=100

Whale Optimization Algorithm (WOA) Models: Grid Search — Reduced Dataset (HbA1c > 8%,9 Features)
==

Model	Best CV Score	Optimal Hyperparameters
AdaBoost	0.8599	algorithm=SAMME, learning_rate=0.8, n_estimators=100
Logistic Regression	0.8959	C=1, max_iter=300, solver=lbfgs
Random Forest	0.9418	max_depth=8, min_samples_split=20, n_estimators=100

Decision Tree	0.8535	max_depth=5, min_samples_split=10
KNN	0.9106	n_neighbors=9, p=1, weights=distance
MLP	0.9408	activation=relu, hidden_layer_sizes=(50,), max_iter=200
Naive Bayes	0.8861	var_smoothing=1e-11
SVM	0.9494	C=1, kernel=linear
XGBoost	0.9376	learning_rate=0.1, max_depth=3, n_estimators=120, subsample=0.9
CatBoost	0.9496	depth=3, iterations=200, learning_rate=0.04
Gradient Boosting	0.9198	learning_rate=0.1, max_depth=2, n_estimators=100

Black Widow Optimization (BWO) Models: Grid Search — Full Dataset (HbA1c >= 6.5%, 12 features)
==

Model	Best CV Score	Optimal Hyperparameters
AdaBoost	0.8340	algorithm=SAMME, learning_rate=1.0, n_estimators=70
Logistic Regression	0.8274	C=10, max_iter=300, solver=lbfgs
Random Forest	0.9086	max_depth=8, min_samples_split=5, n_estimators=100
Decision Tree	0.8171	max_depth=7, min_samples_split=30
KNN	0.8844	n_neighbors=9, p=1, weights=uniform
MLP	0.8916	activation=tanh, hidden_layer_sizes=(50,), max_iter=200
Naive Bayes	0.8268	var_smoothing=1e-11
SVM	0.8697	C=10, kernel=linear
XGBoost	0.8595	learning_rate=0.1, max_depth=7, n_estimators=100, subsample=1.0
CatBoost	0.8758	depth=3, iterations=150, learning_rate=0.1
Gradient Boosting	0.8690	learning_rate=0.05, max_depth=4, n_estimators=100

Black Widow Optimization (BWO) Models: Grid Search — Reduced Dataset (HbA1c > 8%,10 Features
==

Model	Best CV Score	Optimal Hyperparameters
AdaBoost	0.8452	algorithm=SAMME, learning_rate=0.5, n_estimators=70
Logistic Regression	0.8464	C=0.1, max_iter=200, solver=lbfgs
Random Forest	0.9393	max_depth=8, min_samples_split=5, n_estimators=80
Decision Tree	0.8367	max_depth=5, min_samples_split=20
KNN	0.9006	n_neighbors=7, p=2, weights=distance
MLP	0.9165	activation=relu, hidden_layer_sizes=(100,), max_iter=200
Naive Bayes	0.8391	var_smoothing=1e-11
SVM	0.8684	C=1, kernel=linear
XGBoost	0.8804	learning_rate=0.1, max_depth=3, n_estimators=80, subsample=0.8
CatBoost	0.8780	depth=5, iterations=150, learning_rate=0.04
Gradient Boosting	0.8643	learning_rate=0.05, max_depth=2, n_estimators=80
