# Supplementary material for: Predicting visual impairment in type 2 diabetes using key performance indicators identified by the Black Widow and Whale Optimization Algorithms: A case study in Saudi Arabia
Source: PLoS One. 2026 Oct 21;21(10):e0361022. doi: 10.1371/journal.pone.0361022 (PMC13686523; doi:10.1371/journal.pone.0361022)
Supplement: Supplementary file 3 [file pone.0361022.s003.pdf]

**Table S3.** Calibration analysis for the three best-performing models.

| Model | Brier Score | ECE | Train-Test Acc. Gap | Train-Test AUC Gap | Calibration Status |
|---|---|---|---|---|---|
| WOA (CatBoost) | 0.060 | 0.010 | 0.96-0.95=0.01 | 0.98-0.96=0.02 | Excellent |
| BWO (RF) | 0.120 | 0.042 | 0.94-0.92=0.02 | 0.95-0.90=0.05 | Good |
| Baseline (MLP) | 0.100 | 0.022 | 0.95-0.91=0.04 | 0.97-0.94=0.03 | Good |
