# Supplementary material for: Predicting visual impairment in type 2 diabetes using key performance indicators identified by the Black Widow and Whale Optimization Algorithms: A case study in Saudi Arabia
Source: PLoS One. 2026 Oct 21;21(10):e0361022. doi: 10.1371/journal.pone.0361022 (PMC13686523; doi:10.1371/journal.pone.0361022)
Supplement: Supplementary file 4 [file pone.0361022.s004.pdf]

Supplementary Material S1

Black Widow Optimization (BWO): Mathematical Formulation

The Black Widow Optimization (BWO) algorithm is a population-based metaheuristic inspired by the unique mating and survival strategies of black widow spiders. In nature, the reproduction process of these spiders involves mating, procreation, cannibalism, and survival of the fittest, and these behaviours have been mathematically modelled to solve complex optimization problems [1]. Recent studies have further enhanced the original BWO algorithm to improve its optimization capability and feature selection performance across a wide range of applications [2,3].

1. Solution Encoding

The algorithm begins with an initial population of individuals, each representing a candidate solution. For a given optimization problem with N_{var} decision variables, each individual (widow) is expressed as a vector:

$$Widow = \left[x_1, x_2, \dots, x_{N_{var}}\right]$$

In BWO, each solution (widow) is encoded as a binary vector whose length equals the number of features in the dataset. A value of 1 indicates that the feature is selected, while a value of 0 indicates that it is excluded. The quality of each solution is evaluated by the fitness function $f(x)$, which reflects how well the candidate solves the problem [1].

2. Procreation Phase

After initialization, the algorithm proceeds with the procreation phase, where pairs of individuals mate to generate offspring. If x_1 and x_2 are parents, their offspring y_1 and y_2 are produced using a linear combination controlled by a random coefficient α [1]:

$$\begin{cases} y_1 = \alpha * x_1 + (1-\alpha) * x_2 \\ y_2 = \alpha * x_2 + (1-\alpha) * x_1, \end{cases}$$

3. Cannibalism Phase

Following reproduction, the algorithm introduces a cannibalism process, which reflects the natural behaviour of black widow spiders. Weaker individuals are eliminated, leaving only the strongest candidates to advance to the next generation. Cannibalism occurs at three levels: between parents, among siblings, and occasionally between offspring and their mother. This mechanism ensures a natural selection pressure that improves the quality of solutions [1].

4. Mutation Phase

To enhance population diversity, the algorithm incorporates mutation, where a subset of individuals undergoes random modifications by swapping elements within their solution vectors. Mutation is controlled by a predefined mutation rate, which balances exploration and exploitation [1].

5. Algorithm Parameters

The main parameters of BWO are the procreation rate (P_p), cannibalism rate (C_r), and mutation rate (P_m). These parameters regulate the proportion of individuals that reproduce, the percentage eliminated through cannibalism, and the fraction undergoing mutation, respectively [1]. BWO convergence is usually determined by three stopping criteria: reaching a predefined number of iterations, observing no further improvement in the best fitness value over consecutive iterations, or achieving a specified accuracy level. These conditions ensure computational efficiency while maintaining solution quality [1-3].

References

[1] Hu G, Du B, Wang X, Wei G. An enhanced black widow optimization algorithm for feature selection. Knowledge-Based Systems. 2022; 235:107638.
https://www.sciencedirect.com/science/article/pii/S095070512100900X
[2] Abu-Hashem MA, Shehab M, Shambour MK, Daoud MS, Abualigah L. Improved black widow optimization: an investigation into enhancing cloud task scheduling efficiency. Sustainable Computing: Informatics and Systems. 2024; 41:100949.
https://www.sciencedirect.com/science/article/pii/S221053792300104X
[3] Li M, Wang J, Deng S, Zhao Y, Li Y. Enhanced black widow optimization algorithm incorporating food sufficiency strategy and differential mutation strategy for feature selection of high-dimensional data. Expert Systems with Applications. 2025:128506.
https://www.sciencedirect.com/science/article/pii/S0957417425021256
