# Supplementary material for: Predicting visual impairment in type 2 diabetes using key performance indicators identified by the Black Widow and Whale Optimization Algorithms: A case study in Saudi Arabia
Source: PLoS One. 2026 Oct 21;21(10):e0361022. doi: 10.1371/journal.pone.0361022 (PMC13686523; doi:10.1371/journal.pone.0361022)
Supplement: Supplementary file 5 [file pone.0361022.s005.pdf]

Supplementary Material S2

Whale Optimization Algorithm (WOA): Mathematical Formulation

The Whale Optimization Algorithm (WOA) is a bio-inspired metaheuristic optimization algorithm developed to solve complex numerical problems. The algorithm is modeled after the unique hunting behavior of humpback whales, specifically their bubble-net feeding strategy, which reflects two main behavioral phases: encircling prey and spiral-shaped attack. WOA simulates these behaviors to effectively explore and exploit the search space. In nature, humpback whales create a series of bubbles in a spiral shape to trap and catch their prey. Inspired by this process, WOA assumes that the best solution found so far represents the prey, and other candidate solutions (whales) update their positions around it. The algorithm begins by initializing a random population of whales in the search space, and iteratively updates their positions using mathematical models that mimic real whale behavior [1].

1. Encircling Prey Mechanism

The encircling mechanism is represented by the following equations [1]:

$$\vec{D} = \left|\vec{C} \cdot \vec{X}^*(t) - \vec{X}(t)\right| \qquad (1)$$

$$\vec{X}(t+1) = \vec{X}^*(t) - \vec{A} \cdot \vec{D} \qquad (2)$$

Here, $\vec{X}^*(t)$ is the best solution obtained so far, $\vec{X}(t)$ is the current whale's position, and $\vec{A}, \vec{C}$ are coefficient vectors calculated as follows:

$$\vec{A} = 2a \cdot \vec{r} - a \qquad (3)$$

$$\vec{C} = 2 \cdot \vec{r} \qquad (4)$$

where a linearly decreases from 2 to 0 over iterations, and $\vec{r}$ is a random vector in [0,1]. These equations enable whales to converge on the optimal solution by gradually reducing the search radius over time [1].

2. Spiral Updating Mechanism

WOA also features a spiral updating mechanism that simulates the helix-shaped movement of whales toward their prey. This movement is expressed using equation (5):

$$\vec{X}(t+1) = \vec{D}' \cdot e^{bl} \cdot \cos(2\pi l) + \vec{X}^*(t) \qquad (5)$$

where $\vec{D}' = \left|\vec{X}^*(t) - \vec{X}(t)\right|$, b is a constant defining the spiral shape, and l is a random number in $[-1,1]$ [1].

3. Combined Movement Strategy

To model the simultaneous use of both encircling and spiral behavior, the following condition is used:

$$\vec{X}(t+1) = \begin{cases} \vec{X}^*(t) - \vec{A}\cdot\vec{D}, & if\ p < 0.5 \\ \vec{D}'\cdot e^{bl}\cdot\cos(2\pi l) + \vec{X}^*(t), & if\ p \geq 0.5 \end{cases} \quad (6)$$

where p is a random number in [0,1] determining the movement strategy [1].

4. Exploration Phase

For exploration, when the absolute value of vector $\vec{A} > 1$, the algorithm promotes diversification by updating positions relative to a randomly selected whale $\vec{X}_{rand}$, rather than the best-known one. The process follows equations (7) and (8) [1,2]:

$$\vec{D} = \left|\vec{C}\cdot\vec{X}_{rand}(t) - \vec{X}(t)\right| \quad (7)$$

$$\vec{X}(t+1) = \vec{X}_{rand}(t) - \vec{A}\cdot\vec{D} \quad (8)$$

This helps the algorithm escape local optima and explore new regions of the search space [2].

WOA successfully balances exploration (searching for new solutions) and exploitation (refining known good solutions) through this dual mechanism, and has shown strong performance in applications such as feature selection, data classification, and engineering optimization [2,3].

References

[1] Mirjalili S, editor. Handbook of Whale Optimization Algorithm: Variants, Hybrids, Improvements, and Applications. Elsevier; 2023. https://books.google.com.au/books?hl=en&lr=&id=si2gEAAAQBAJ&oi=fnd&pg=PP1&dq=%5B53%5D+S.+Mirjalili,+%E2%80%9CWhale+optimization+algorithm,%E2%80%9D+in+Handbook+of+Metaheuristics,+Springer,+2021.&ots=eOqi2sXrb8&sig=QYlLolPiDMn5zgKLkXb5c5xIyxM&redir_esc=y#v=onepage&q=%5B53%5D%20S.%20Mirjalili%2C%20%E2%80%9CWhale%20optimization%20algorithm%2C%E2%80%9D%20in%20Handbook%20of%20Metaheuristics%2C%20Springer%2C%202021.&f=false
[2] Rana N, Latiff MS, Abdulhamid SI, Chiroma H. Whale optimization algorithm: a systematic review of contemporary applications, modifications and developments. Neural Computing and Applications. 2020;32(20):16245-77. https://link.springer.com/article/10.1007/s00521-020-04849-z
[3] Guo W, Liu T, Dai F, Xu P. An Improved Whale Optimization Algorithm for Feature Selection. Computers, Materials & Continua. 2020;62(1). https://www.techscience.com/cmc/v62n1/38116
